# Substrate Specificity of a Methyltransferase Involved in the Biosynthesis of the Lantibiotic Cacaoidin

**DOI:** 10.1021/acs.biochem.4c00150

**Published:** 2024-09-13

**Authors:** Haoqian Liang, Youran Luo, Wilfred A. van der Donk

**Affiliations:** † Department of Biochemistry, University of Illinois at Urbana−Champaign, Urbana, Illinois 61801, United States; ‡ Department of Chemistry and Howard Hughes Medical Institute, University of Illinois at Urbana−Champaign, Urbana, Illinois 61801, United States; § Carl R. Woese Institute for Genomic Biology, University of Illinois at Urbana−Champaign, Urbana, Illinois 61801, United States

## Abstract

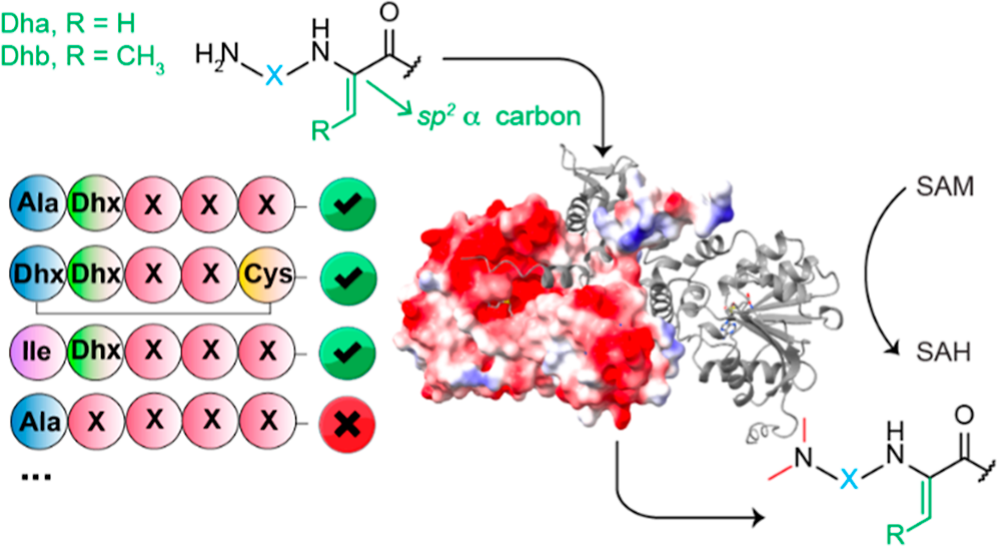

Modification of the N- and C-termini of peptides enhances their stability against degradation by exopeptidases. The biosynthetic pathways of many peptidic natural products feature enzymatic modification of their termini, and these enzymes may represent a valuable pool of biocatalysts. The lantibiotic cacaoidin carries an *N*,*N*-dimethylated N-terminal amine group. Its biosynthetic gene cluster encodes the putative methyltransferase Cao4. In this work, we present reconstitution of the activity of the enzyme, which we termed CaoS_C_ following standardized lanthipeptide nomenclature, using a heterologously produced peptide as the model substrate. In vitro methylation of diverse lanthipeptides revealed the substrate requirements of CaoS_C_. The enzyme accepts peptides of varying lengths and C-terminal sequences but requires dehydroalanine or dehydrobutyrine at the second position. CaoS_C_-mediated dimethylation of natural lantibiotics resulted in modestly enhanced antimicrobial activity of the lantibiotic haloduracin compared to that of the native compound. Improved activity and/or metabolic stability as a result of methylation illustrates the potential future application of CaoS_C_ in the bioengineering of therapeutic peptides.

## Introduction

Natural products possess significant value in various applications.^1^ They serve not only as a primary source of therapeutic agents but also offer a wide array of potential biocatalysts given the diversity of enzymatic reactions involved in their biosynthetic pathways.^2,3^ Ribosomally synthesized and post-translationally modified peptides (RiPPs) represent an increasingly diverse category of natural products that are characterized by the enzymatic modification of genetically encoded precursor peptides.^4,5^ The first RiPP biosynthetic enzymes that were biochemically characterized were the proteins involved in the biosynthesis of microcin B17 in a pioneering study by Walsh and co-workers.^6^ Since then, the field has rapidly expanded and now encompasses more than 45 different natural product families.^7^ Precursor peptides of RiPPs typically consist of two segments, an N-terminal leader peptide (LP) that contributes to the binding affinity for many of the enzymes involved in post-translational modifications (PTMs) and a C-terminal core peptide (CP) where the PTMs take place.^7^ The PTMs confer RiPPs with extensive structural diversity, endowing these compounds with a wide spectrum of bioactivities.^8−10^ One type of modification that is widely found in many different RiPP families is N-terminal methylation. Methyl transfer in nature is catalyzed by methyltransferases with the largest family using *S*-adenosyl methionine (SAM) as the cosubstrate.^11−14^ In this study, we characterized one such RiPP methyltransferase, investigated its predicted structure and substrate selectivity, and placed it in context with other RiPP methyltransferases.

The largest family of currently known RiPPs is the lanthipeptides, which are characterized by the β-thioether cross-linked bis-amino acids lanthionine (Lan) and methyllanthionine (MeLan).^15^ Lanthipeptides are subdivided into five classes depending on differences in protein structure and mechanism employed by the (Me)Lan synthetases.^15−21^ Beyond the characteristic thioether rings, lanthipeptides often contain additional tailoring modifications that expand their structural diversity.^15,22^ These tailoring modifications may bestow on the lanthipeptides higher affinity and selectivity for their targets by additional molecular interactions and/or contribute to peptide stability.^7,15,22^ Biochemical studies of these tailoring reactions may facilitate use of the enzymes involved for peptide bioengineering and synthetic biology applications.^23^

**Figure 1:**
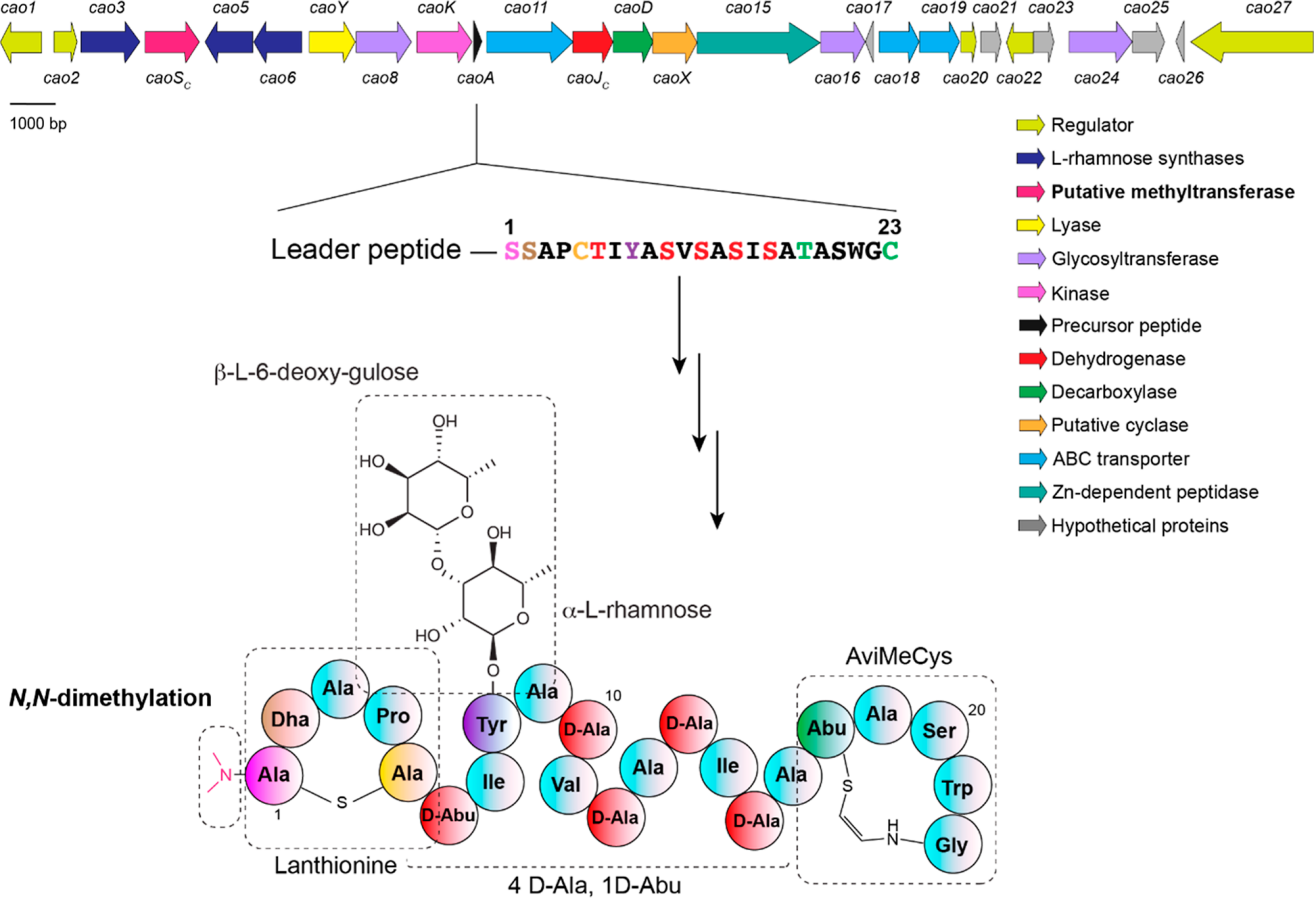
Sequence and structure of cacaoidin. The sequence of the CaoA CP is depicted under the BGC. The residues that undergo PTMs are colored the same in the CP and the structure of mature cacaoidin. Cacaoidin combines various structural characteristics in one molecule, including an *N*,*N*-dimethyl lanthionine, five d-amino acids, an *O*-glycosylated tyrosine, and a C-terminal aminovinyl-methyl-cysteine (AviMeCys). The enzymes encoded in the *cao* BGC are depicted in colors that correspond to the modifications they install on the CaoA peptide when such information is available or has been proposed. Abu, 2-aminobutyric acid.

Cacaoidin is the founding member of the class V lanthipeptides and is produced by *Streptomyces cacaoi* CA-170360 (Figure 1).^19,24^ Cacaoidin exhibits potent antimicrobial activity against Gram-positive bacteria including methicillin-resistant *Staphylococcus aureus*.^5,25^ The compound combines various structural characteristics in one molecule, including a dimethylated N-terminal Lan (Figure 1).^19,24^ N-Terminal α-N-methylation is frequently found in RiPPs including linaridins,^26−31^ phomopsin,^32,33^ linear azol(in)e-containing peptides,^34^ the class II lanthipeptides divamide A and archalan,^35,36^ the class III lanthipeptides andalusicin, bacinapeptin, and paenithopeptin,^37,38^ and the class V lanthipeptides lexapeptide and pristinin A3.^20,21^ N-Methylation is believed to improve the metabolic stability of peptides owing to decreased aminopeptidase susceptibility and higher lipophilicity.^39^ Understanding the mechanism and substrate requirement of the enzyme that catalyzes the dimethylation of cacaoidin could potentially benefit future bioengineering of peptides.

An initial sequence-based bioinformatics analysis showed the presence of a gene encoding a putative methyltransferase in the *cao* biosynthetic gene cluster (BGC) (Figure 1).^19^ The current study reports the in vitro activity of this proposed methyltransferase, demonstrating that it catalyzes α-*N*,*N*-dimethylation on the N-terminal amine of substrate peptides. The nomenclature of lanthipeptide biosynthetic enzymes follows a common naming convention with the generic prefix Lan followed by a specific letter depending on the type of enzymatic activity.^40^ Following this standardized naming scheme, lanthipeptide methyltransferases have been designated LanS with the methyltransferases that convert l-Asp into l-isoAsp through *O*-methylation of the carboxylate side chain termed LanS_A_, whereas the term LanS_B_ was introduced for methyltransferases that methylate the C-terminal carboxylate of lanthipeptides.^7,41,42^ Following the use of LanS as the generic name for methyltransferases in lanthipeptide gene clusters, we suggest naming the enzyme in the *cao* BGC CaoS_C_ and the α-*N*-methyltransferase family involved in lanthipeptide biosynthesis LanS_C_. In this work, the substrate selectivity of CaoS_C_ was investigated through bioinformatic sequence analysis of putative substrates coupled with in vitro methylation of heterologously produced lanthipeptides. Potential bioactivity changes of the lantibiotic analogues that resulted from CaoS_C_ dimethylation were also examined.

## Materials and Methods

### General Methods and Reagents

Primers and gBlocks were ordered through TWIST Bioscience and Integrated DNA Technologies. Factor-Xa, Gibson Assembly Master Mix, and Q5 HF DNA Polymerase were purchased from New England Biolabs. Alkylation reagent NEM (*N*-ethylmaleimide), reducing reagent tris(2-carboxyethyl)phosphine (TCEP), methyl group donor SAM, and nisin were purchased from Sigma-Aldrich. Super DHB for use as matrix in MALDI-TOF MS was purchased from Sigma-Aldrich. The 4–20% precast polyacrylamide gels used for sodium dodecyl-sulfate polyacrylamide gel electrophoresis (SDS-PAGE) were procured from Bio-Rad. Prestained Rec Protein ladder and Brain Heart Infusion media were purchased from Fisher Scientific. DNA miniprep, PCR purification, and gel extraction kits were obtained from Qiagen. The Amicon centrifugal filters used for protein and peptide concentrations were manufactured by EMD Millipore. PCR was performed using a Bio-Rad C1000 thermocycler. HisTrap resins and columns for immobilized metal affinity chromatography purification of protein and peptide were purchased from GE Healthcare. Protein purification was conducted using an Amersham Biosciences KTApurifier fast protein liquid chromatography (FPLC) system purchased from GE Healthcare. Size exclusion chromatography (SEC) purification was conducted using SuperdexTM 75 10/300 GL resin. Peptide was purified using an Agilent 1260 Infinity II high-performance liquid chromatography (HPLC) system. Cytolysin and variants were purified using a Phenomenex Jupiter Proteo column (4 μm, 90 Å, 10 × 250 mm) using 0.1% trifluoroacetic acid (TFA) in H_2_O (solvent A) with 0.1% TFA in CH_3_CN (solvent B) running at 4 mL/min. Haloduracin and synthetic peptides were purified using a Macherey-Nagel NUCLEODUR C18 HTec column (5 μm, 100 Å, 10 × 250 mm) using the same solvent phases as those used for cytolysin. High-resolution mass spectrometry (HRMS) was performed on an Agilent 6545 liquid chromatography electrospray ionization-quadrupole-time-of-flight (LC-ESI-QTOF) mass spectrometer with a Kinetex 2.6 μm C8 100 Å LC column (150 × 2.1 mm). HRMS data were analyzed using Agilent MassHunter. Matrix-assisted laser desorption/ionization time-of-flight mass spectrometry (MALDI-TOF MS) analysis was carried out in the mass spectrometry facility at UIUC using a Bruker Daltonics UltrafleXtreme mass spectrometer. MALDI-TOF MS data were calibrated using Protein Calibration Standard I purchased from Bruker and processed using the software FlexAnalysis. AlphaFold2 structure predictions were performed with Google Colab and analyzed with Pymol or Chimera X.^43−48^ For bioactivity assays, M17 broth was purchased from Sigma-Aldrich. Glucose was purchased from Fisher Scientific. OD_600_ was measured on a Synergy H4 Hybrid Multi-Mode Microplate Reader from BioTek.

### Cloning

All primers and gBlocks used for cloning and sequencing are listed in Table S1 (Supporting Information). A synthetic gene encoding CaoS_C_ (formerly named *cao4*) codon optimized for expression in *Escherichia coli* was ordered from TWIST Bioscience (Table S1). The gene encoding His_6_–CaoS_C_ was cloned into pRSFDuet-1 multiple cloning site I (MCSI) by PCR followed by Gibson assembly. pRSFDuet-1-His_6_–CylA (27–412),^49^ pRSFDuet-1-His_6_–CylL_S_ (MCSI)-CylM (MCSII),^50^ pRSFDuet-1-His_6_–CylL_L_ (MCSI)-CylM (MCSII),^50^ pRSFDuet-1-His_6_–CylL_S_-(T1S/T2S) (MCSI)-CylM (MCSII),^51^ pRSFDuet-1-His_6_–CylL_S_-(T1S/T2A) (MCSI)-CylM (MCSII),^51^ pRSFDuet-1-His_6_–CylL_S_-(T1A) (MCSI)-CylM (MCSII),^52^ pRSFDuet-1-His_6_–CylL_S_-(T2A) (MCSI)-CylM (MCSII),^51^ pRSFDuet-1-His_6_–Xa-HalA1 (MCSI)-HalM1 (MCSII),^53^ and pRSFDuet-1-His_6_–Xa-HalA2 (MCSI)-HalM2 (MCSII)^53^ were adopted from previously reported studies. For His_6_–CylL_S_-(T1S/T2S/P3A/A4P), a synthetic gene codon-optimized for *E. coli* expression was obtained (see Supporting Information) and amplified by PCR. The plasmid backbone was also amplified by PCR using pRSFDuet-1-His_6_–CylL_S_-(T1S/T2S) (MCSI)-CylM (MCSII) as the template, and the two pieces were assembled into one plasmid through Gibson assembly. *E. coli* DH10B was used as the host for plasmid propagation, and DNA sequencing to confirm successful cloning was completed by ACGT, Inc. For MCSI, sequencing was completed by using ACYCDuetUP1 and DuetDOWN1 primers supplied by ACGT, Inc. For MCSII, sequencing was completed with DuetUP2 and T7 term primers.

### Expression and Purification

*E. coli* NovaBlue T1^R^ Singles competent cells transformed with the pRSFDuet-1 plasmid described above were inoculated in 10 mL of LB with 50 μg/mL kanamycin. This small-scale culture was used to inoculate 1 L of LB media containing 50 μg/mL kanamycin. The cultures were grown at 37 °C with shaking at 220 rpm to an O.D. at 600 nm of 0.6, and overexpression was induced by addition of IPTG to 1 mM final concentration. After 18 h continued shaking at 18 °C, the cultures were centrifuged for 40 min at 4000*g* at 4 °C.

For protein expression and purification, the cell pellet was resuspended in protein lysis buffer (20 mM Tris–HCl, 0.5 M NaCl, 10% glycerol, pH 7.5), lysed by sonication (35% amplitude, 5 min, 2.0 s pulse on, 5.0 s pulse off), and then centrifuged at 49,900*g* for 30 min at 4 °C. The supernatant was loaded on a HisTrap column and eluted using an FPLC instrument with protein elution buffer (20 mM Tris–HCl, 0.5 M NaCl, 10% glycerol, 1 M imidazole, pH 7.5). The elution fractions were collected, concentrated, and loaded for SEC purification using protein storage buffer (20 mM HEPES, 500 mM KCl, 10% glycerol, pH 7.5). The elution fractions corresponding to the molecular mass of the desired protein (determined by SDS-PAGE) were collected and concentrated to 0.5 mM. The concentration was determined on a NanoDrop based on the 280 nm absorption using the extinction coefficient predicted by Expasy. For gel analysis, protein elution buffer containing 0.5 μg of protein was diluted with 4× SDS loading buffer. The sample was boiled at 90 °C for 8 min and then loaded in the gel. The gel was run for 50 min at 180 V in 1× Tris glycine SDS buffer, and the protein bands were visualized by Coomassie blue staining. The protein sample was then flash-frozen and stored at −80 °C.

For peptide purification, cell pellet was resuspended in lysis buffer (6 M guanidine HCl, 20 mM NaH_2_PO_4_, 500 mM NaCl, 0.5 mM imidazole, pH 7.5) and lysed by sonication (75% amplitude, 5 min, 2.0 s pulse on, 5.0 s pulse off) and then centrifuged at 49,900*g* for 30 min at 4 °C. The supernatant was loaded on 2 mL of packed Ni-NTA His Bind Resin in lysis buffer. The resin was washed with wash buffer (4 M guanidine HCl, 20 mM NaH_2_PO_4_, 300 mM NaCl, 30 mM imidazole, pH 7.5), and peptides were eluted in peptide elution buffer (4 M guanidine HCl, 20 mM Tris–HCl, 100 mM NaCl, 1 M imidazole, pH 7.5). The elution fraction was collected and concentrated. To obtain the CP of cytolysin S (CylL_S_″) and cytolysin L (CylL_L_″), recombinant CylA protease was expressed and purified as previously described.^49^ The peptide solution was treated with CylA (0.1 mg/mL) in a 100:1 ratio overnight at ambient temperature. The digested peptide solution was acidified with the addition of 2% TFA, centrifuged at 4500 rpm for 10 min, filtered through a 0.45 μm polyvinylidene fluoride (PVDF) syringe filter, and subjected to HPLC purification. To obtain haloduracin α (Halα) and haloduracin β (Halβ), the post-translationally modified peptides HalA1 and HalA2 were treated with Factor Xa (0.1 mg/mL) in a 100:1 ratio, followed by a similar purification procedure as described for cytolysin. The purified peptide was mixed with 1 μL of saturated Super DHB in 1:1 MeCN/H_2_O and 1 μL was spotted on a MALDI plate. MALDI-TOF MS analysis was conducted on a Bruker UltrafleXtreme mass spectrometer in reflection mode with a 900–4500 Da range. The sample was then lyophilized and stored at −20 °C.

### NEM Assay

A two-step reaction was conducted for NEM analysis, including a reduction reaction and a subsequent alkylation reaction. The reduction reaction was set up in 20 μL, including 0.5 mg/mL target peptide, 50 mM phosphate buffer (pH = 5.5), and 5 mM TCEP. The reaction was kept at room temperature for 1 h to reduce all potential disulfide linkages in the peptide samples. Subsequent addition of 500 mM Tris buffer (pH = 8) adjusted the pH of the reaction mixture to 7.5, to which NEM was added to a final concentration of 5 mM. The alkylation reaction was allowed to proceed at room temperature for 30 min before analysis by MALDI-TOF MS. Parallel reactions using unmodified His_6_–CylL_S_-(T1S/T2S/P3A/A4P) were always performed as a positive control.

### In Vitro Methylation

For methylation, the 50 μL assay contained 50 μM peptide, 5 μM His_6_–CaoS_C_, and 1 mM SAM in 20 mM HEPES buffer (pH 7.5). After overnight reaction at 37 °C, the proteins were denatured by adding 5 μL of 2% TFA and centrifuged for 10 min at 4500 rpm in a microcentrifuge at room temperature. The supernatant was desalted using an EMD Millipore C18 ziptip, and the peptide was eluted and analyzed by MALDI-TOF MS as described above.

To obtain sufficient amounts of dimethylated peptide for the bioactivity assay, the same methylation assay was successfully scaled up to 10 mL in one tube. After overnight reaction at 37 °C, the proteins were denatured by adding 1 mL of 2% TFA, and the sample was centrifuged for 2 min at 14,000*g* at room temperature. The supernatant was subjected to HPLC purification. The purified peptide was analyzed by MALDI-TOF MS as described above. The sample was then lyophilized and stored at −20 °C.

### Preparation of Haloduracin β

*Bacillus halodurans* C-125 was grown on Brain Heart Infusion agar plates (15 mm × 150 mm) for 120 h at 30 °C. Bacteria were harvested from the agar plates via scraping and resuspended in 70% isopropyl alcohol in sterile deionized water (v/v). The cell suspension was incubated for 24 h at 30 °C with vigorous agitation. Cells and debris were removed by centrifugation at 49,900*g* for 30 min at 4 °C, and the haloduracin-containing supernatant was concentrated via rotary evaporation. The resultant supernatant was filtered using a 0.45 μm PVDF syringe filter to remove any residual cells. Then, 10 mM TCEP solution was added into the supernatant to reduce the disulfide bond formed in Halα. This step is important for differentiating the retention time of Halα and Halβ in a HPLC system, which facilitates peptide separation.^53^ After 1 h incubation with TCEP, the solution was injected into an HPLC system, and the peptides were purified following the same procedure as described above.

### Kinetic Assay of Methylation of Haloduracin β

The initial rates of methylation of haloduracin β were monitored by LC–MS adopted from a previously reported method.^54^ All data points were collected in triplicate. Reaction quench buffer was prepared (1.1 M citric acid and 5.5 mM EDTA), of which 3 μL was added into 0.2 mL Eppendorf tubes. The enzymatic assay consisted of a 30 μL reaction that contained 1 mM SAM and varying concentrations of haloduracin β (20, 40, 60, 80, 100, 150, and 200 μM) in CaoS_C_ reaction buffer (20 mM HEPES, pH = 7.5). The mixture was incubated at room temperature for 10 min, and His_6_-CaoS_C_ was added to reach a final concentration of 0.5 μM. Aliquots were taken from the reaction vial and added to the Eppendorf tubes containing quench solution at set time points. The quenched samples were analyzed by LC–MS using an Agilent LC–MS qTOF instrument with a Kinetex 2.6 μm C8 100 Å, LC column (150 × 2.1 mm). The column was eluted with solvent A (100% H_2_O, 0.1% formic acid) and solvent B (100% acetonitrile, 0.1% formic acid) at a flow rate of 0.40 mL/min. The elution gradient was 30%–60% solvent B over 6 min. The qTOF instrument settings were as follows: ion mode = positive, ion source = Dual AJS ESI, gas temperature = 200 °C, drying gas = 13 L/min, nebulizer = 35 psi, sheath gas temperature = 350 °C, sheath gas flow = 11 L/min, and MS TOF fragmentor = 125 V. Data were collected over an *m*/*z* range of 50–3000 Da in continuous mode with a 0.2 s scan rate, using the LC–MS qTOF reference solution kit as an internal calibration standard. For each substrate concentration, initial rates were determined for reactions that had progressed to <20% conversion by integration of the peak for the substrate ion. Linearity of the ion count was observed in serial dilutions of a stock solution of haloduracin β at the concentrations used in the assays (Supporting Information).

### Antibacterial Assay for MIC Determination

MICs were determined in 96-well microtiter plates. GM17 media (M17 + 0.5% glucose) was applied, and 10 μL overnight cultures of *Lactococcus lactis* sp. cremoris were used to inoculate 1 mL of subculture. When the OD_600_ of the subculture reached 0.5, the subculture was diluted to 10^5^ CFU/mL. In each well, 100 μL of cell suspensions was added, followed by 100 μL of each peptide dissolved in GM17 media. The peptides were serially diluted to reach final concentrations of 256, 128, 64, 32, 16, 8, 4, and 2 nM. The cells were incubated at 30 °C for 16 h, and OD_600_ was measured on a Synergy H4 Hybrid Multi-Mode Microplate Reader.

### LC-ESI-QTOF Analysis of Methylated Peptides

After the methylation reaction, the sample was loaded onto a Glygen TopTip C-18 and eluted from the TopTip with 100 μL of 0.1% formic acid in MeCN. Elution buffer was then lyophilized and dissolved in 20 μL of HPLC grade water, injected on a Kinetex C8 LC column, and analyzed in positive detection mode on an Agilent 6545 LC-ESI-QTOF with a drying gas temperature of 320 °C and gas flow of 13 L/min and a sheath gas temperature of 350 °C and gas flow of 11 L/min. All mass spectrometry data were deposited at https://data.mendeley.com/datasets/45vf5k5dfv/1.

## Results

### Reconstitution of CaoS_C_ Activity Using a Heterologously Produced Lanthipeptide

In the *lxm* BGC involved in the biosynthesis of the class V lanthipeptide lexapeptide, *lxmM* encodes a methyltransferase homologue to the experimentally characterized cypemycin α-*N*,*N*-methyltransferase CypM (32% sequence identity to LxmM with 72% coverage).^20,27,28^ Single gene deletion of *lxmM* resulted in the production of demethyl-dehydro-lexapeptide, suggesting that LxmM is responsible for the *N*,*N*-dimethylation of Phe1 in lexapeptide. Similar to lexapeptide and cypemycin, cacaoidin is also *N*,*N*-dimethylated on its N-terminal Lan.^19,24^ The *cao4* gene in its BGC encodes a putative methyltransferase CaoS_C_ that is annotated as a putative *O*-methyltransferase in the NCBI protein database. CaoS_C_ does not display sequence similarity to either LxmM or CypM, implying that CaoS_C_ may be a representative of a group of RiPP *N*-methyl transferases that has not been investigated previously.

**Figure 2:**
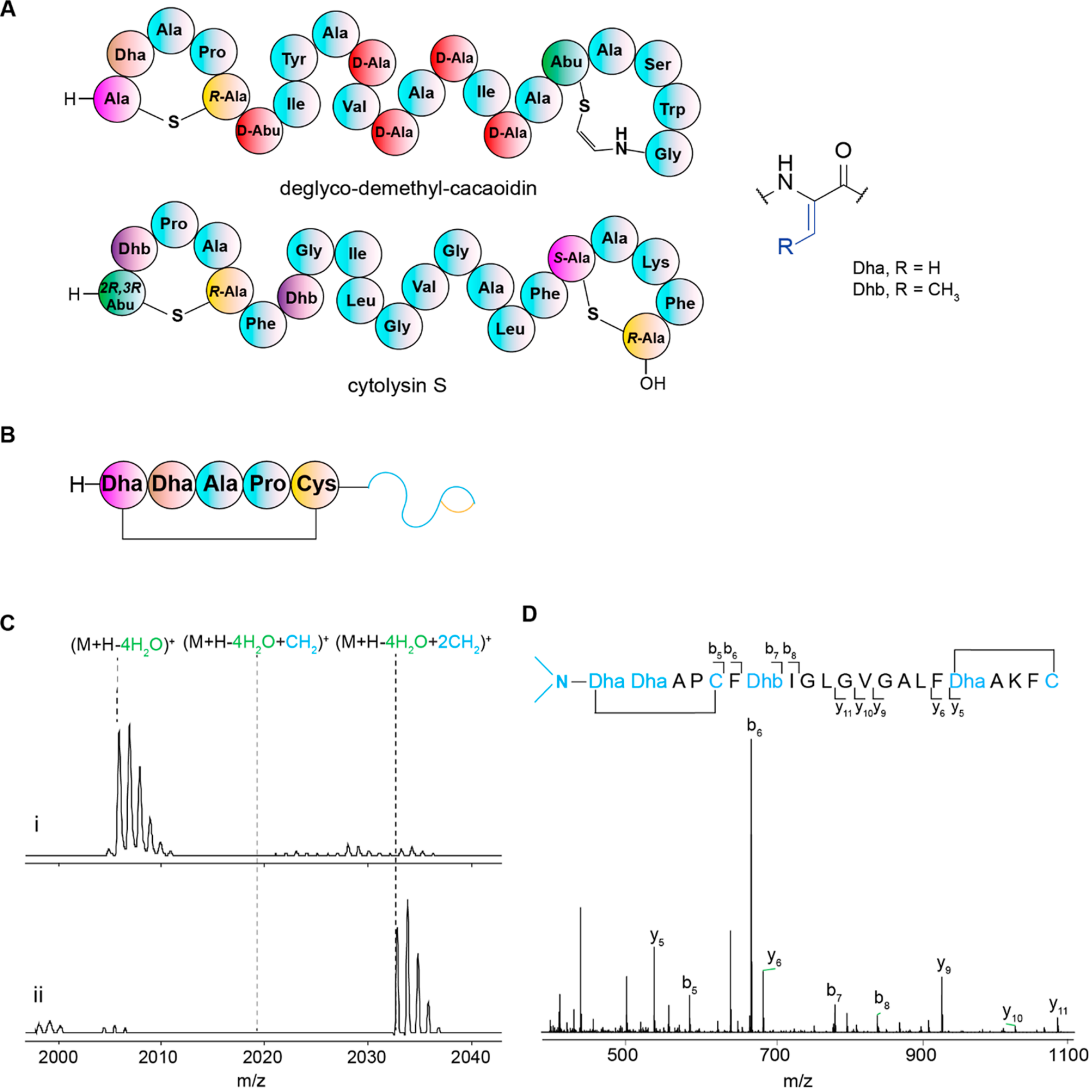
(A) Structure comparison between deglyco-demethyl-cacaoidin and cytolysin S. The stereochemistry of the N-terminal Lan in cacaoidin is not known. Also shown are the structures of the α,β-unsaturated amino acids Dha and Dhb generated by the dehydration of Ser/Thr. (B) Schematic representation of CylM-modified, CylA-digested His_6_–CylL_S_-(T1S/T2S/P3A/A4P). The N-terminal sequence of the peptide is described in detail, with a short-hand depiction of the C-terminal structure that is identical with wild-type cytolysin S in panel A. (C) MALDI-TOF MS of CylM-modified CylL_S_-(T1S/T2S/P3A/A4P) CP before (i) and after CaoS_C_ treatment in the presence of SAM (ii). In (i), (M + H – 4H_2_O)^+^ calcd. *m*/*z* = 2004.0, obsd. *m*/*z* = 2003.7. In (ii), (M + H – 4H_2_O + CH_2_)^+^ calcd. *m*/*z* = 2018.0; (M + H – 4H_2_O + 2 CH_2_)^+^ calcd. *m*/*z* = 2032.0, obsd. *m*/*z* = 2032.2. (D) LC-ESI-QTOF MS–MS fragmentation pattern of dimethylated CylM-modified CylL_S_-(T1S/T2S/P3A/A4P) CP generated by CaoS_C_. For fragment masses, see Table S2.

Methyltransferases that install N-terminal methyl groups on a lanthipeptide require a CP without the N-terminal LP. Because of the high hydrophobicity and poor ionization of the CaoA CP sequence (Figure 1),^55^ neither the unmodified CaoA CP nor the modified CP could be purified by HPLC after LP removal. This poor solubility is consistent with the reported target of cacaoidin, the membrane-bound lipid II,^25^ and suggests that one of the functions of diglycosylation may be increasing its solubility. To still investigate the substrate selectivity of the methyltransferase, a class II lanthipeptide cytolysin S (CylL_S_″)^56^ was adopted as a model substrate in this study. CylL_S_″ shares high similarity in both sequence and structure with deglyco-demethyl-cacaoidin in the N-terminal segment of the peptides where the dimethylation occurs, and it exhibits higher hydrophilicity and better ionization probably owing to the Lys near the C-terminus (Figure 2A).^57^ CylL_S_″ contains an N-terminal five-amino acid MeLan-containing ring formed from the sequence Dhb1-Dhb2-Pro3-Ala4-Cys5 (Dhb = dehydrobutyrine formed by dehydration of Thr) (Figure 2A). Cacaoidin possesses a similar N-terminal five-amino acid ring but with two dehydroalanine (Dha) substitutions of the two Dhb residues in the ring precursor and exchange in position of the Pro and Ala residues. Both rings originate from a Dhx1-Dhx2-Xxx3-Xxx4-Cys5 pattern (Dhx = Dha or Dhb) (Figure 2A). This similarity made CylL_S_″ a promising substrate candidate for CaoS_C_ since the methyltransferase was expected to act after (Me)Lan formation and was anticipated to mainly recognize the N-terminal peptide sequence.^15^

To determine its activity, His_6_-tagged CaoS_C_ was expressed and purified from *E. coli*, forming a homodimer based on analytical SEC analysis. To generate a cytolysin analogue that would be an even closer cacaoidin mimic, a variant of the precursor peptide CylL_S_, His_6_–CylL_S_-(T1S/T2S/P3A/A4P), was coexpressed with the cytolysin synthetase CylM in *E. coli*, followed by LP removal by its dedicated serine protease CylA in vitro^49,57^ and HPLC purification (Figure 2B). Analysis by MALDI-TOF MS demonstrated four dehydrations of Ser and Thr residues, and treatment with the Cys alkylating reagent *N*-ethylmaleimide showed the absence of free thiols, suggesting correct cyclization (Figure S1A),^58^ which was also supported by tandem MS (Figures 2D and S1C). The methylation reaction was then performed in vitro using purified His_6_–CaoS_C_, the CP of CylM-modified CylL_S_-(T1S/T2S/P3A/A4P) and SAM. CaoS_C_ successfully added two methyl groups on the peptide (Figure 2C). Methyltransferases often exhibit strong product inhibition by *S*-adenosyl-l-homocysteine (SAH),^59^ requiring inclusion of SAH hydrolase to obtain full conversion in assays, but CaoS_C_ activity did not appear to be strongly inhibited. The modification pattern of the in vitro reaction product was examined by tandem HRMS, demonstrating that the two methyl groups were installed at the amine group of the N-terminal Lan, similar to mature cacaoidin generated by the native producer (Figure 2D, Table S2). The tandem MS analysis also verified the sites of dehydration and cyclization. Incubation of CylL_S_-(T1S/T2S/P3A/A4P) with CaoS_C_ for long duration (24 h) even resulted in peptide with a third methyl group on the N-terminus, as demonstrated by neutral loss of the resultant trimethylammonium group in electrospray ionization-quadrupole-time-of-flight-mass spectrometry (LC-ESI-QTOF) (Figure S1B–D, Table S3).^60^ Collectively, these results revealed the α-*N*-methyltransferase activity of CaoS_C_. The successful dimethylation of CylM-modified CylL_S_-(T1S/T2S/P3A/A4P) illustrates that methylation can occur prior to other modifications during cacaoidin biosynthesis, such as glycosylation and AviMeCys formation, which is an important observation with respect to utilization of the enzyme as a general N-terminal methyltransferase. However, at present, we cannot rule out that the enzyme can also methylate desmethylcacoidin that already has all other modifications installed.

### Predicted Structure of CaoS_C_

**Figure 3:**
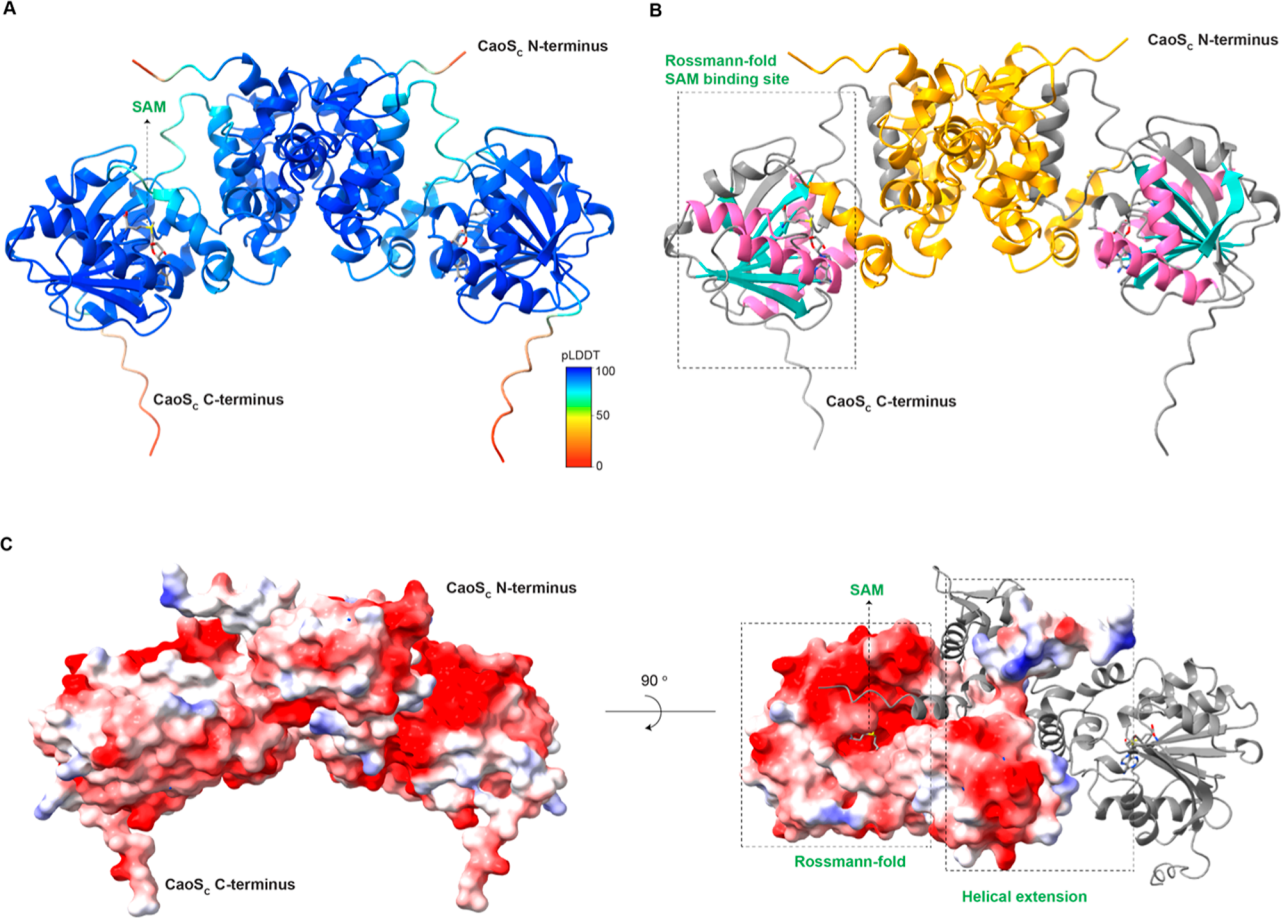
(A) Overall structure of the rank_1 model from AlphaFold2 prediction of the CaoS_C_ homodimer along with the methyl group donor SAM. CaoS_C_ is colored by the pLDDT values. (B) Structure of the Rossmann-fold SAM binding site in the predicted model. The classic four α-helices are depicted in pink, and six β-sheets are in blue. The N-terminal extension of CaoS_C_ is colored orange, which likely participates in peptide substrate binding. (C) Surface analysis of the predicted CaoS_C_ homodimer, with a negatively charged surface in red and a positively charged surface in navy.

AlphaFold2-Multimer was used to generate a predicted structural model of the CaoS_C_ homodimer.^46^ The model is of high confidence, as indicated by the predicted local distance difference test (pLDDT) values (Figure 3A). In the predicted structure, the two monomers interact with each other through a helical N-terminal domain. The CaoS_C_ C-terminus forms the classic Rossmann fold with the SAM binding site surrounded by multiple α-helixes (Figure 3B).^61^ Natural product methyltransferases contain a wide array of extensions to the Rossman domain, reflecting the high structural diversity of their substrates. CaoS_C_ is predicted to have a peptide substrate binding extension that is mostly helical (Figure 3B). The highly negatively charged surface of CaoS_C_ (Figure 3C) is somewhat surprising as cacaoidin does not have any positively charged residues other than its N-terminus (Figure 1). It is possible that CaoS_C_ electrostatically interacts with other biosynthetic enzymes during the biosynthesis of cacaoidin. The predicted structure of the CaoS_C_ monomer was also generated using AlphaFold2 (Figure S2),^48^ which aligns well with the protomer of the CaoS_C_ homodimer.

### CaoS_C_ Belongs to a RiPP Methyltransferase Family

The similarity in catalytic function but the distinction in sequence between CaoS_C_ and LxmM prompted a phylogenetic investigation. Methyltransferases involved in natural product biosynthesis are a very diverse family of enzymes.^59^ Based on fold, they typically fall into one of two superfamilies (class I and class III).^59,62^ Currently known methyltransferases involved in RiPP biosynthesis comprise a relatively small group of 17 enzymes consisting mostly of members of the class I methyltransferase (MT) family containing the Rossman fold for SAM binding along with diverse substrate recognition domains. A phylogenetic tree reflects their strong diversity, illustrating a number of different protein families involved in RiPP methylation (Figure S3). CaoS_C_ is a member of the class I methyltransferases and falls into an *O*-methyltransferase protein family (PF00891) with other characterized methyltransferases from different RiPP families, none of which catalyze methylation of the N-terminus.^63,64^ CaoS_C_ has a relatively close relationship with StspM (40% sequence identity to CaoS_C_ with 85% coverage), a methyltransferase that catalyzes C-terminal O-methylation of a lasso peptide.^65^ AlphaFold2 models show that both StspM and CaoS_C_ are homologues of the crystallographically characterized neocarzinostatin *O*-methyltransferase NcsB1 (PDB ID: 3I53, Figure S4)^66^ and also resemble the polyketide antibiotic rhodomycin hydroxylase RdmB (PDB ID: 1XDS; dimer).^67,68^ The helical N-terminal extensions in NcsB1 and RdmB are likely involved in small molecule binding. CaoS_C_ is also closely related to a putative methyltransferase SprMe (46% sequence identity to CaoS_C_ with 93% coverage) that is involved in the biosynthesis of another class V lanthipeptide pristinin A3, but this enzyme has not yet been experimentally characterized (Figure S4).^21^

The RiPP α-*N*-methyltransferases LxmM and CypM are members of the *N*-methyltransferase CcbJ family (class I methyltransferase, PDB ID: 4HGY) that have a very different fold of their substrate recognition domain than CaoS_C_ based on AlphaFold2 predictions (Figure S5).^20,28,69^ The tree in Figure S3 shows that LxmM, CypM, and CaoS_C_ are quite distantly related in sequence to other α-*N*-methyltransferases in RiPPs, such as BamL/BpumL involved in the biosynthesis of the poly azol(in)e-containing peptide plantazolicin,^70−74^ PhomM that is expected to function in the biosynthesis of the fungal RiPP phomopsin,^33^ DivMT involved in trimethylation during the biosynthesis of the class II lanthipeptide divamide,^35^ and AncMT and PttMT from the BGCs of the class III lanthipeptides andalusicin and paenithopeptin (Figures S5 and S6).^37,38^ A predicted model of AncMT shows a substrate binding fold similar to that of CypM/LxmM even though they appear evolutionarily distantly related (AncMT has 22% sequence identity to LxmM with 40% coverage) (Figure S5). The *O*-methyltransferases BmbA,^75^ AgeMT,^76^ OlvS_A_,^41^ LahS_B_,^42^ and the *N*-methyltransferase LegD all belong to the class I methyltransferases, but they have structurally diverse substrate recognition domains (Figure S6).^30^ Notably, the crystallographically characterized OphMA that methylates the amide backbone in borosins is a class III methyltransferase that lacks the Rossman-fold domain.^59,77−79^ The methyltransferase AlnMT encoded in the BGC of the archaeal class II lanthipeptide archalan, which was proposed to catalyze monomethylation on the N-terminus of archalan β,^36^ surprisingly belongs to the radical SAM protein family (closest structural homologue 4-demethylwyosine synthase).^80,81^ From a chemical reactivity perspective, N-methylation is not expected to require radical chemistry, although at least one example has been reported of a radical SAM enzyme that catalyzes nonradical, S_N_2-type methylation.^82^ The predicted structure (Figure S6I) and the presence of the expected ligands for two iron–sulfur clusters support the assignment of a radical SAM enzyme, but its involvement in N-methylation requires experimental confirmation. Regardless, RiPP methyltransferases appear to be highly diverse with respect to their mechanisms of peptide substrate recognition.

### CaoS_C_ Requires a Planar Residue at Position 2 in the Substrate

**Figure 4:**
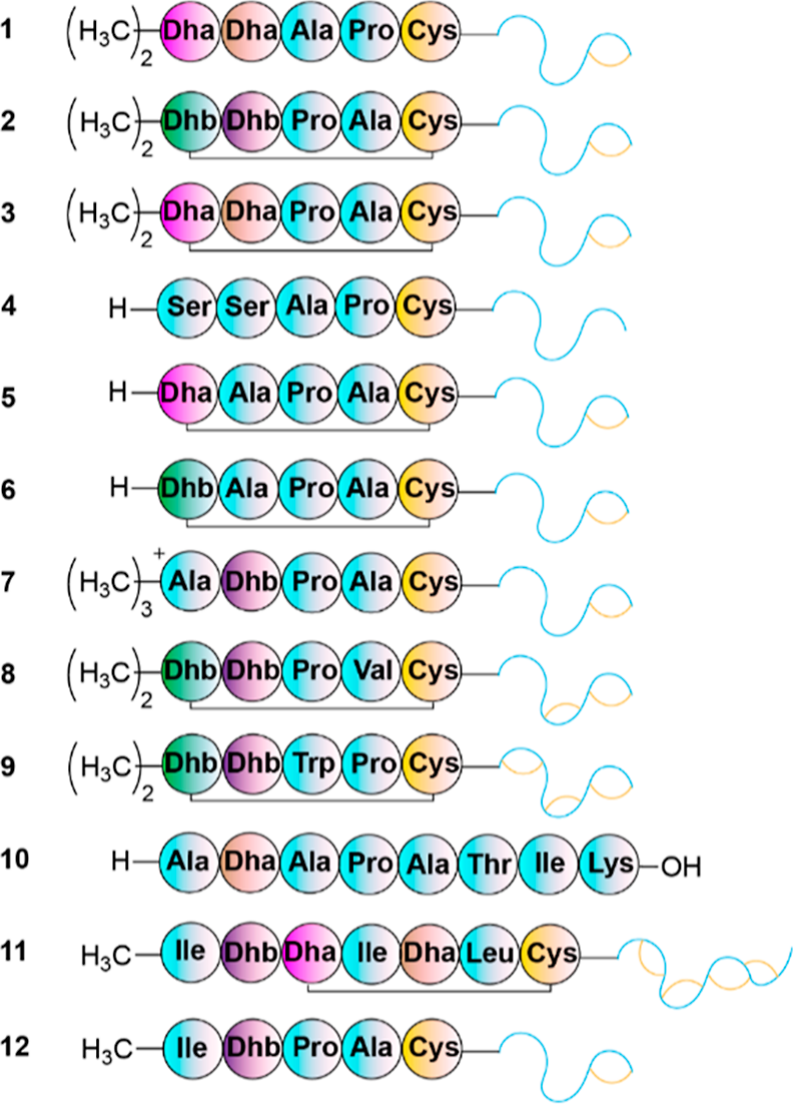
Summary of CaoS_C_ activity with all of the peptides evaluated in this study. Peptides are represented schematically. The N-terminal sequence of the peptide is described in detail, with a short-hand depiction of the C-terminal structure. Peptides that were substrates are shown as methylated to different extents.

The substrate requirement of CaoS_C_ was probed next. Dimethylation of CylL_S_-(T1S/T2S/P3A/A4P) by CaoS_C_ not only reflected tolerance of the enzyme regarding the C-terminal substrate sequence but also ruled out many of the PTMs on mature cacaoidin being required for CaoS_C_ to recognize the substrate, including the d-amino acids, *O*-glycosylated tyrosine carrying a disaccharide, and the AviMeCys motif. To examine the role of the sequence inside the N-terminal five-amino acid ring in substrate recognition, mature wild-type CylL_S_″ was selected for CaoS_C_ treatment in vitro. Despite the differences with cacaoidin (Figure 2A), this peptide was also dimethylated by CaoS_C_ (entry 2, Figures 4 and S7). This result indicates that rather than sequence, CaoS_C_ might require other structural characteristics in the substrate, which could be either the thioether bridge containing five amino acids, the dehydroamino acid at the second position of the CP, or a combination of these two features. The same conclusion was also drawn from CaoS_C_ activity toward another variant, CylL_S_-(T1S/T2S) modified by CylM. In this peptide, the first two amino acids are Lan and Dha to resemble cacaoidin, but the Pro and Ala positions are switched compared to cacaoidin. Dimethylation of CylL_S_-(T1S/T2S) was again observed (entry 3, Figures 4 and S8).

To further determine the structural features that dictate CaoS_C_ activity, more CylL_S_ variants were made and subjected to CaoS_C_ treatment. To first investigate whether any posttranslational modifications within the N-terminal five-amino acid sequence of CaoA were required, unmodified CylL_S_-(T1S/T2S/P3A/A4P) was produced in *E. coli*, followed by CylA cleavage to remove the LP. This unmodified peptide was then treated with CaoS_C_ and SAM, and methylation was not observed after 24 h (entry 4, Figures 4 and S9), suggesting the necessity of either dehydration or cyclization or both (Figure S9). Dehydration at position 1 appears unlikely as a requirement because an uncyclized Dha residue at the N-terminus of the CP would spontaneously hydrolyze into a ketone after LP removal.^83,84^ Such a hydrolyzed substrate would no longer contain an N-terminal amino group to be methylated. The dehydroamino acid at the second position of the CP was therefore targeted next. CylL_S_-(T1S/T2A) and CylL_S_-(T2A) were dehydrated and cyclized by CylM, and the products maintained the N-terminal rings generated from Dhx-Ala-Pro-Ala-Cys. Surprisingly, no methylation was observed on these peptides after overnight treatment with CaoS_C_, suggesting the importance of Dhx2 rather than the thioether ring in substrate selectivity (entries 5 and 6, Figures 4 and S10, S11).

The importance of Dhx2 was supported in methylation reactions with CylL_S_ variants that lacked a Lan/MeLan ring but retained Dhx2. Initially, CylM-modified and CylA-cleaved CylL_S_-(T1A) was used, resulting in three dehydrations and one uncyclized Cys, as expected since Thr1 can no longer be dehydrated and cyclized (Figure S12B). Reaction of this peptide that lacks an N-terminal ring but retains Dhb2 with CaoS_C_ resulted in three methylations on the N-terminus of the peptide (entry 7, Figures 4 and S12C,D, Table S4). Together with the previous results, CaoS_C_ appears to have a strong preference for an amino acid with sp^2^ hybridization of the α and β carbons, such as dehydroalanine or dehydrobutyrine, at the second position in the substrate. A detailed molecular explanation of this preference will require a cocrystal structure of substrate-bound CaoS_C_, but despite much effort, the enzyme has proven recalcitrant to structure elucidation. Presumably, the linear (noncyclized) flexible peptide with Dhx at the second position (entry 7, Figure 4) also allowed a third methylation, whereas the Lan/MeLan rings in the other substrate peptides evaluated here disfavored formation of a conformation facilitating such a third methylation.

### CaoS_C_ Exhibits Substrate Tolerance toward Other Lanthipeptides

A bioinformatic analysis of putative substrates of CaoS_C_ orthologues was performed next to provide additional potential insight into its substrate requirement. Among BGCs with co-occurrence of genes encoding LanK, LanY, and LanS_C_, peptides shorter than 110 residues and containing Cys and Ser/Thr in the C-terminal region were collected and aligned.^5,85,86^ The collected peptides mirrored the findings from previous multiple sequence alignment of putative type A class V lanthipeptides (Figure S13),^55^ revealing strong sequence conservation within the C-terminal region of the predicted LP as well as the N-terminal sequence of the predicted CP. In the latter, Thr1 was observed in nearly all peptides with Ser or Ala substitution in some sequences. These three residues (after CylM modification) were all accepted in vitro by CaoS_C_ for methylation in the various peptides tested in this study. Notably, Dhx in the second position of the CP (Ser/Thr before CylM modification) was fully conserved, which is consistent with the strong preference of CaoS_C_ for Dha/Dhb at this position in our experimental data (Figure 4). Of course, the strong conservation at this position could also be related to the requirements for antimicrobial activity of cacaoidin, but structure–activity relationship information regarding this position is currently not available.

**Figure 5:**
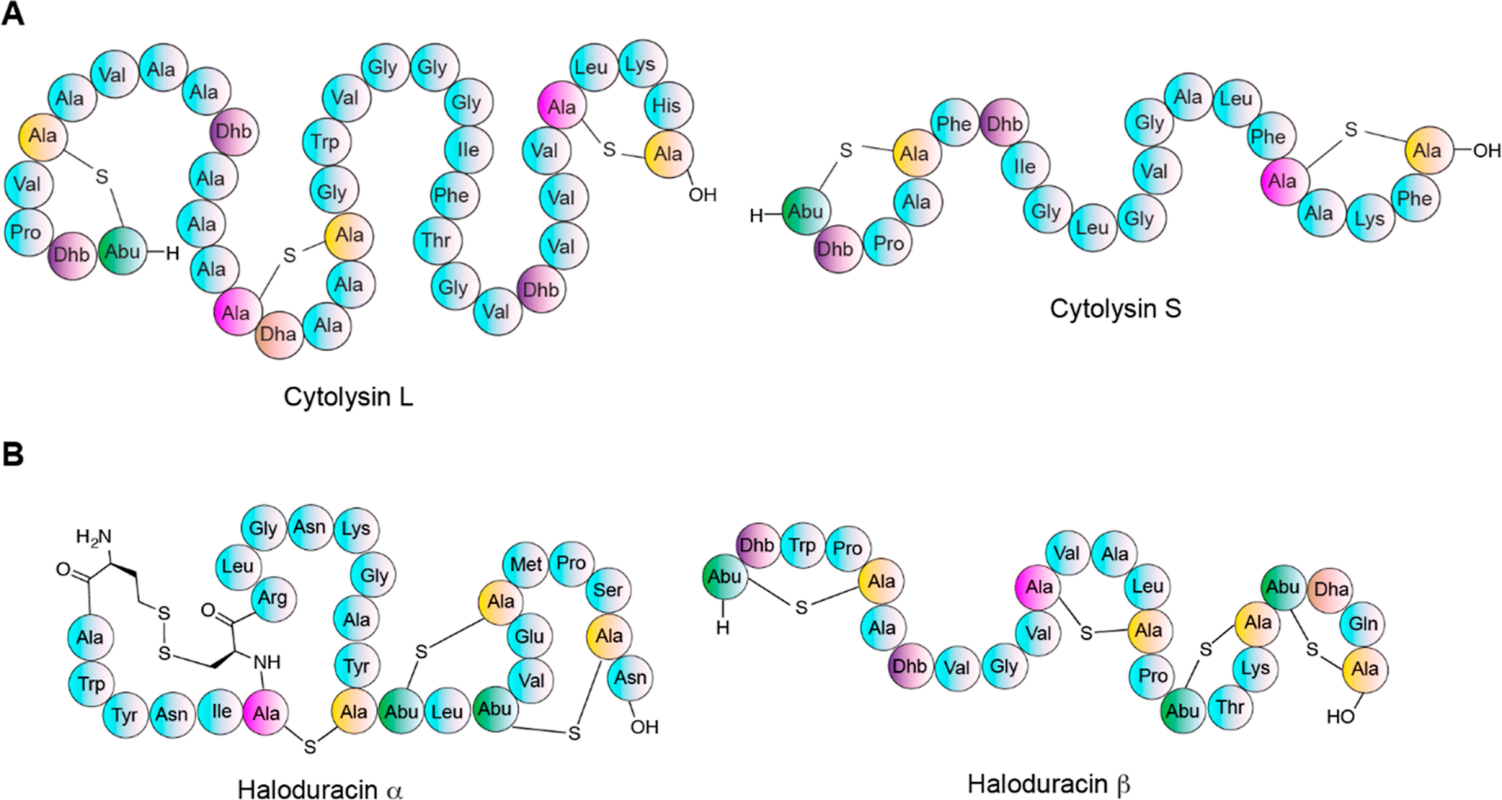
Schematic representation of the two-component lanthipeptides cytolysin (A) and haloduracin (B).

With additional knowledge from the conservation of residues in natural substrates, the tolerance of CaoS_C_ for application in lantibiotic bioengineering was investigated. The class II lanthipeptide cytolysin L (CylL_L_″) has 38 amino acids, including seven dehydroamino acids and three thioether bridges and shares some structural similarity with cacaoidin at the N-terminal region (Figure 5A). The compound was incubated with CaoS_C_ and SAM, and two methyl groups were introduced on the N-terminus of the peptide (entry 8, Figures 4 and S14, Table S5).^56^ CaoS_C_ also dimethylated another class II lanthipeptide haloduracin β (Halβ), which again has a similar five-amino acid N-terminal ring but contains a large amino acid Trp right after Dhb2 (entry 9, Figures 4, 5B, and S15, Table S6).^87^ We also attempted to methylate a short peptide with a dehydroamino acid at position 2, but a synthetic 8-residue peptide was not accepted (Ala-Dha-Ala-Pro-Ala-Thr-Ile-Lys, Figure S16). Given the activity observed with uncyclized CylL_S_″ variants with a similar N-terminal sequence (entry 10, Figure 4), the enzyme appears to need a longer sequence to provide sufficient affinity for catalysis.

To test the hypothesis that Dhx2 in a peptide substrate is important for CaoS_C_ activity, the class I lanthipeptide nisin that shares no sequence homology with cacaoidin but does have Dhb at the second position was evaluated next.^88^ Nisin contains three dehydroamino acids and five Lan/MeLan rings with a total length of 34 residues. Incubation of nisin with CaoS_C_ resulted in monomethylated nisin, with the methylation confirmed to be located at the N-terminal Ile1 by LC-ESI-QTOF MS–MS (entry 11, Figures 4 and S17, Table S7). We surmised that the single methylation observed with nisin might be because of the steric requirements of the Ile side chain at position 1. To test this hypothesis, we made a mutant of the substrate in entry 7 that was methylated three times by replacing Ala at position 1 with Ile. Parallel experiments with the peptides in entry 7 and entry 12 showed that indeed at time points where the peptide in entry 7 was trimethylated, the Ile analogue in entry 12 was only methylated once (Figure S18, Table S8). Attempts to methylate haloduracin α (Halα) by CaoS_C_ were unsuccessful, which might be explained by the absence of a dehydroamino acid in position 2 (Figure 5B).

### Dimethylation of Lanthipeptides Alters Bioactivity

N-Methylation is a well-known strategy for enhancing the stability and bioavailability of peptide drug candidates.^89^ Successful incorporation of methyl groups onto peptides such as CylL_L_″ and CylL_S_″ by CaoS_C_ motivated investigation into a potential corresponding bioactivity change. The class II lanthipeptide cytolysin is a two-component lanthipeptide produced by *Enterococcus faecalis* that serves as a virulence factor.^90^ Cytolysin is made up of subunits L and S (Figure 5A), which work together in a 1:1 ratio to lyse both bacterial and mammalian cells.^91^ Cytolysin is potent, with MICs in the nanomolar range against many Gram-positive bacteria.

Dimethylated CylL_L_″ and CylL_S_″ were obtained via CaoS_C_ treatment, followed by HPLC purification. Antibacterial activity assays were performed against the indicator strain *L. lactis* sp. cremoris in GM17 liquid growth media. Wild-type cytolysin exhibited an MIC < 2 nM. Using cytolysin subunits that were both dimethylated resulted in inhibition of bacterial growth at 32 nM (Tables 1, S9, and S10). The higher MIC of the dimethylated peptide might be due, in part, to the observed poor solubility of the methylated peptides. Alternatively, *N*,*N*-dimethylation may have modulated the conformation of the peptide(s), potentially leading to decreased affinity for a target molecule or for its partner peptide. Combination of *N*,*N*-diMe-CylL_L_″ with wild-type CylL_S_″ also resulted in significantly increased MIC (32 nM). Conversely, only a 2-fold increase in the MIC compared to that of wild type was observed when *N*,*N*-diMe-CylL_S_″ was combined with wild-type CylL_L_″ (MIC = 4 nM), indicating that dimethylation had a more negative impact on CylL_L_″, possibly related to its poor solubility (Tables 1, S11, and S12).

**Table 1:** Bioactivity Profile of CaoS_C_-Dimethylated Lantibiotics and Wild-Type Lantibiotics

peptide^a^	MIC (nM)^b^	peptide^a^	MIC (nM)^b^
CylL_L_″ + CylL_S_″	<2	DiMe-CylL_L_″ + CylL_S_″	32
CylL_L_″ + DiMe-CylL_S_″	4	DiMe-CylL_L_″ + DiMe-CylL_S_″	32
Halα + Halβ	16	Halα + DiMe-Halβ	8

a Two subunits of peptides were combined in a 1:1 ratio.

b Minimum inhibitory concentration against *L. lactis* sp. cremoris.

The change in the MIC as a result of dimethylation of the two-component class II lantibiotic haloduracin was also assessed. Haloduracin is composed of α and β subunits and has high potency against a range of Gram-positive bacteria (Figure 5B).^92^ Similar to cytolysin, the two subunits of haloduracin display optimal activity in a 1:1 stoichiometry.^92^ Mode of action studies revealed that Halα binds to lipid II to prevent peptidoglycan biosynthesis, while cationic Halβ interacts with the complex of lipid II-Halα causing the transient formation of pore-like structures in the cell membrane.^93^*N*,*N*-Dimethylated Halβ displayed high solubility in liquid media. A bioactivity assay using *L. lactis* sp. cremoris as the indicator strain demonstrated a 2-fold lower MIC for dimethylated Halβ combined with nonmethylated Halα compared to the combination of native compounds, indicating modestly increased potency brought by dimethylation (Tables 1, S13, and S14).

### Kinetic Analysis of Methylation of Haloduracin β

The demonstration that CaoS_C_ was able to methylate haloduracin β also provided an opportunity to quantitatively investigate this process because this lanthipeptide is much more soluble than cytolysin or cacaoidin. We used a discontinuous LC–MS assay to measure initial rates of the first methylation event by measuring the disappearance of the substrate at a series of substrate concentrations up to 200 μM, which is nearing the solubility limit under the conditions used, and a fixed concentration of 1 mM SAM. A fit of the initial rates to the Michaelis–Menten equation provided a *K*_m_ of 61 ± 6 μM for haloduracin β and a *k*_cat_ of 3.1 ± 0.1 min^–1^ (Figure S19), values that are on par with the activity of lanthipeptide synthases and other natural product biosynthetic enzymes.^58^

## Discussion

In this study, we assign the function of the methyltransferase CaoS_C_ encoded in the *cao* BGC. Because of the insolubility of modified CaoA, the activity of CaoS_C_ was reconstituted using a variant of the class II lanthipeptide CylL_S_″ that resembles cacaoidin at its N-terminus. In vitro experiments revealed CaoS_C_ to be an α-*N*-methyltransferase that catalyzes *N*,*N*-dimethylation of the N-terminal amine group. CaoS_C_ catalyzes a similar modification as another class V lanthipeptide methyltransferase LxmM and the linaridin methyltransferase CypM,^20,28,29^ but CaoS_C_ is distinguished from these proteins as both sequence and predicted structure analysis demonstrated that CaoS_C_ falls into a different methyltransferase family. In general, characterized RiPP methyltransferases are very diverse in structure with the peptide substrate binding domains showing sequence and structural similarity to enzymes from non-RiPP natural product or cofactor biosynthetic enzymes. It appears that these enzymes were recruited from diverse pathways and that their chemoselectivity (*O*- vs *N*-methyltransferase activity) diverged.

The substrate tolerance of the enzyme was investigated by in vitro methylation assays with multiple peptides, suggesting that CaoS_C_ requires residues that have a dehydroamino acid, such as Dha or Dhb, at the second position in the substrate. This requirement was also suggested through a multiple sequence alignment of the putative native substrate peptides based on the co-occurrence of LanKYS_C_ and in vitro methylation tests on diverse lanthipeptides.^94^ Interestingly, a recent genome mining study on class III lanthipeptides identified a large number of BGCs that show co-occurrence of a methyltransferase and precursor peptides with a Ser/Thr at position 2.^38^ Analysis of the substrate selectivity of one of the methyltransferases (PttMT) in a heterologous host led to the conclusion that Ala-Dhx and Ala–Ala were preferred substrates. However, the predicted structure of PttMT differs from that of CaoS_C_ (Figure S5) and is more similar to another class III lanthipeptide methyltransferase AncMT.

Bioactivity changes as a result of dimethylation catalyzed by CaoS_C_ were examined with two lantibiotics. For the two-component lantibiotic cytolysin, both components were dimethylated, but a decrease in potency was observed in MIC determination assays, possibly due to the poor solubility of the dimethylated peptides in the cell culture media. For another two-component lantibiotic haloduracin, only the β subunit could be dimethylated, and the resultant peptide displayed slightly enhanced antimicrobial activity when combined with the wild-type partner α subunit. This example shows that CaoS_C_ can be deployed for generating new-to-nature lantibiotics. Furthermore, the enzyme as well as other RiPP N-terminal methyltransferases may be used for improving the stability of peptide therapeutics.

## Supporting Information Available

AlphaFold structures, mass spectrometric data, and phylogenetic analyses; primer and gene sequences; masses of observed ions; and OD_600_ values of *L. lactis* sp. cremoris growing in GM17 media with lantibiotics (PDF)
