# Supplementary material for: Substrate Specificity of a Methyltransferase Involved in the Biosynthesis of the Lantibiotic Cacaoidin
Source: Biochemistry. 2024 Sep 13;63(19):2493–505. doi: 10.1021/acs.biochem.4c00150 (PMC11447909; doi:10.1021/acs.biochem.4c00150)
Supplement: Supplementary file 1 — bi4c00150_si_001.pdf [file bi4c00150_si_001.pdf]

Supporting information

Substrate specificity of a methyltransferase involved in the biosynthesis of the lantibiotic cacaoidin

Haoqian Liang[1], Youran Luo[2] and Wilfred A. van der Donk[1-3*]

[1] Department of Biochemistry University of Illinois at Urbana-Champaign, Urbana, Illinois 61801, USA. [2] Department of Chemistry and Howard Hughes Medical Institute, University of Illinois at Urbana-Champaign, Urbana, Illinois 61801, USA. [3] Carl R. Woese Institute for Genomic Biology, University of Illinois at Urbana-Champaign, Urbana, Illinois 61801, USA.

* To whom correspondence should be addressed:

Wilfred A. van der Donk

600 S. Mathews Avenue

Urbana, Illinois 61801, United States

vddonk@illinois.edu

phone: (217) 244-5360

fax: (217) 244-8533

Materials and Methods

Synthesis of Ala1-Dha2-Ala3-Pro4-Ala5-Thr6-Ile7-Lys8

The peptide was synthesized following a general procedure[1] using NovaPEG Rink Amide LL resin loaded with Fmoc-protected Lys at 0.1 mmol scale. The synthesized product was then cleaved from the resin and globally deprotected in the cleavage cocktail (95:5:5 of TFA/H_2O/triisopropylsilane). After HPLC purification, the peptide was treated with 2,5-dibromohexanediamide as reported previously.[2] Conversion of Cys to Dha was confirmed by MALDI-TOF MS analysis. The product was purified by HPLC and subjected to $CaoS_C$ treatment.

Table S1. List of primers and double stranded DNA fragments.

DNA Name	Sequence
Cao4_Optimized_Seq	ATGGCGACGGAAATTCCCCCGCAAGTGCGTGATTTGC<br>TTAAGAAGGCTGATCTTTTACCACCTACCGCCATTCGT<br>ATTGCTGCCACTCTCCGTATTGCGGATCATATTGCGGA<br>TGGTGTGCGTGATCCTGCCGGAATTGCCGAACGTGCG<br>GGAGCTCAGCGTGTTCCTATGGAACGTTTGTTACGTTA<br>CTTAGCAACGATTGATGTTTTATGTGAAACAGATGAT<br>GGTGGATATGATGTGACGGGCACGGGTGCATTATTAC<br>GTTCTGGCGCGGGTTTACGTGAAGCACTTGATATTGAT<br>GGTTTGATGGGTCGTGGTGAAAGTGCTACCCCTTTTCT<br>TTTACATACCTTGCGTACGGGAAAGAGTGCATATGCG<br>GGTGTTCATGGGCGTGAATATTGGGAAGATGTGAATG<br>CGGAACCCGTATTTCGTGAACAACTTAAGGCGCTTGG<br>TCATTCAGGCTTAGCATGGGAAGGCGAACGTATTGTG<br>AAGGAATATCCTTGGTCAGGTGTGGGACATGTGGTGG<br>ATGTGGGTGGGAATAAGGGTCAACTCCTTGTGGCTCT<br>CCTCAGTGAATTTTCGCACCTTCGTGGTACGTTGGTGG<br>AATTACCTAATTTAGTTGAACTTGCGACGGAAGATTTT<br>GCGCGTGCTGGTCTCCAAGATCGTTGTACGGCCGTAG<br>TTGGAGATTTCTTTGCTGAGCTTCCCGCAGGTGCAGAC<br>GTTTATGTGTTGTCAGGTGTGTTGGCGGATTGGGATGA<br>TGAAGATGCCGTTCGTATTCTTCGTCGTTGTGCGGAAG<br>CTTGTGGCACCCGTGGGCGTATTTTGTTAGCAGATATT<br>GACCTTGTTCTCGATATGCCCGACCCCGCAGATCAAG<br>CTGCCGCAGAGTTACGTACGATGGCTACGGTTCCTGG<br>AATTGGTCGTGATGTGGATGGTGTAAAGGAGTTAGCG<br>GGACGTGCGGGCCTTGATATTACCTGGGAAGGAGAAG<br>CATCAGCGATTCGTTCTTTATTGGAACTCACCCCACGT<br>ACGGGTACGGCACAAGCCGCGCCCGATGGCGGCGCTT<br>AA
LL_CylLs_SSAP_F1	GGTCTGGGTGTTGGTGCAC
LL_CylLs_SSAP_R1	CATCACCGCTACCCTGAATTGC
LL_CylLs_SSAP_Insert	GCAATTCAGGGTAGCGGTGATGTTCAGGCAGAAAGCA<br>GCGCACCGTGTTTTACCATTGGTCTGGGTGTTGGTGCA<br>C
LL_CylLs_core_SSAP_F1	CATCACCATCATCACCACAGCC
LL_CylLs_core_SSAP_R1	CTGTTCGACTTAAGCATTATGCGGC
LL_CylM_Dh_F1	GTAATCGTATTGTACACGGCCGCATAATC
LL_CylM_Dh_R1	CGAAAACGCGGGAAAAAGTGGAAG
LL_CylLs(SSAP)_F1	CTTCCACTTTTTCCCGCGTTTTCG

LL_CylLs(SSAP)_R1	GATTATGCGGCCGTGTACAATACGATTAC
LL_CylLs_SAPAC_Inser	GCAATTCAGGGTAGCGGTGATGTTCAGGCAGAAAGCG CACCGGCATGCTTTACCATTGGTCTGGGTGTTGGTGCA C
LL_CylLs_ASAPA_F1	GTGGGGTGCATCCGGC
LL_CylLs_ASAPA_R1	GTGCTAACTGCATCGTGAAGAATGACC
LL_SAPAAA_BB_F1	GCGGCCGCATAATGCTTAAGTC
LL_SAPAAA_BB_R1	CATGCCGGTGCGGATTCTG
LL_SAPAAA_BB_Inser	GTTCAGGCAGAATCCGCACCGGCATGTTTTACCATTG GTCTGGGTGTTGGTGCACTGTTTGCAGCAAAATTTGCA TAAGCGGCCGCATAATGCTTAAG
LL_SAPAAA_BB_F1	GCGGCCGCATAATGCTTAAGTC
LL_SSAPAA_BB_R1	GCCGGATTGGAAGTACAGGTTC
LL_SSAPAA_BB_Inser	GTTCAGGCAGAAAGCAGCGCACCGTGTTTTACCATTG GTCTGGGTGTTGGTGCACTGTTTGCAGCAAAATTTGCA TAAGCGGCCGCATAATGCTTAAG
YR_CylLs_T1I-F1	CAGGCAGAAATTACACCGGCATGTTTTAC
YR_CylLs_T1I-R	AACATCACCGCTACCCTGAATTGC

Table S2. Calculated ppm error of the fragment ions observed in the tandem mass spectrum of dimethylated CylL$_S$-(T1S/T2S/P3A/A4P) shown in Figure 1.

Mass fragment	Calcd. m/z	Obsd. m/z	ppm error
$[M+3H]^{3+}$	678.0067	678.0031	5.31
y_{11}^+	1081.5499	1081.5498	0.09
y_{10}^+	1024.5284	1024.5304	-1.95
y_9^+	925.4600	925.4611	-1.19
y_6^+	684.3174	684.3181	-1.02
y_5^+	537.2490	537.2496	-1.12
b_5^+	585.2490	585.2495	-0.85
b_6^+	668.2861	668.2867	-0.90
b_7^+	781.3702	781.3711	-1.15
b_8^+	838.3916	838.3917	-0.12

Table S3. Calculated ppm error of the fragment ions observed in the tandem mass spectrum of trimethylated CylL$_S$-(T1S/T2S/P3A/A4P) shown in Figure S1.

Mass fragment	Calcd. m/z	Obsd. m/z	ppm error
$[M+2H]^{2+}$ [a]	1023.5206	1023.5205	0.10
y_{21}^+	993.9839	993.9830	0.91
y_{15}^+	1447.7766	1447.7745	1.45
y_{14}^+	1364.7395	1364.7362	2.42
y_{13}^+	1251.6554	1251.6533	1.68
y_{11}^+	1081.5499	1081.5476	2.13
y_9^+	925.4600	925.4576	2.59
y_7^+	797.4015	797.4004	1.38
y_6^+	684.3174	684.3163	1.61
y_5^+	537.2490	537.2475	2.79
b_5^+	393.1227	393.1221	1.53
b_7^+	623.2282	623.2274	1.28

b_8^+	736.3123	736.3114	1.22
b_9^+	793.3338	793.3317	2.65

[a] MS^1 of trimethylated CylL$_S$-(T1S/T2S/P3A/A4P). All the listed MS^2 are generated from this trimethylated CylL$_S$-(T1S/T2S/P3A/A4P) precursor after the neutral loss of N-terminal trimethylamine.

Table S4. Calculated ppm error of the fragment ions observed in the tandem mass spectrum of trimethylated CylL$_S$-(T1A) shown in Figure S11.

Mass fragment	Calcd. m/z	Obsd. m/z	ppm error
$[M+2H]^{2+}$	1031.5325	1031.5404	-7.66
y_{11}^+	1081.5499	1081.5387	10.4
y_9^+	925.4600	925.4657	-6.16
y_5^+	537.2490	537.2515	-4.65
b_1^+	197.1285	197.1285	0
b_4^+	468.2275	468.2259	3.42
b_6^+	698.3330	698.3355	-3.58
b_7^+	811.4171	811.4125	5.67
b_8^+	868.4386	868.4374	1.38
b_{10}^+	1038.5441	1038.5466	-2.41

Table S5. Calculated ppm error of the fragment ions observed in the tandem mass spectrum of dimethylated cytolysin L shown in Figure S13.

Mass fragment	Calcd. m/z	Obsd. m/z	ppm error
$[M+3H]^{3+}$	1155.5858	1155.5832	2.25
y_{29}^+	1330.1672	1330.1623	3.68
y_{28}^+	1288.6487	1288.6447	3.10
y_{27}^+	1253.1301	1253.1326	-2.00
y_7^+	767.4233	767.4246	-1.70
b_5^+	494.2432	494.2418	2.83
b_6^+	565.2803	565.2808	-0.90
b_7^+	664.3487	664.3469	2.71
b_8^+	735.3858	735.3850	1.09
b_9^+	806.4229	806.4229	0.00
b_{10}^+	889.4600	889.4595	0.56

Table S6. Calculated ppm error of the fragment ions observed in the tandem mass spectrum of dimethylated Halβ shown in Figure S14.

Mass fragment	Calcd. m/z	Obsd. m/z	ppm error
$[M+2H]^{2+}$	1180.0464	1180.0437	2.29
y_{17}^{+}	1624.7624	1624.7575	3.02
y_{16}^{+}	1525.6960	1525.6923	2.43
b_{5}^{+}	581.2541	581.2540	0.17
b_{6}^{+}	652.2912	652.2906	0.92
b_{7}^{+}	735.3283	735.3283	0.00
b_{8}^{+}	834.3967	834.3960	0.84

Table S7. Calculated ppm error of the fragment ions observed in the tandem mass spectrum of mono-methylated nisin shown in Figure S16.

Mass fragment	Calcd. m/z	Obsd. m/z	ppm error
$[M+3H]^{3+}$	1038.4588	1038.4594	-0.58
y_{33}^{+}	1620.2378	1620.2387	-0.56
y_{4}^{+}	452.2616	452.2600	3.54
b_{30}^{+}	1458.1605	1458.1529	5.21
b_{31}^{+}	1018.1291	1018.1259	3.14
b_{32}^{+}	1051.1519	1051.1473	4.38
b_{33}^{+}	1074.1591	1074.1600	-0.84

Table S8. Calculated ppm error of the fragment ions observed in the tandem mass spectrum of mono-methylated $CylL_S$-(T1I) shown in Figure S18.

Mass fragment	Calcd. m/z	Obsd. m/z	ppm error
$[M+2H]^{2+}$	1038.5441	1038.5419	-2.10
y_{14}^{+}	1364.7395	1364.7385	-0.74
y_{11}^{+}	1081.5499	1081.5490	-0.85
y_{9}^{+}	925.4600	925.4596	-0.24
y_{7}^{+}	797.4014	797.3992	-2.79
y_{6}^{+}	684.3174	684.3170	-0.64
y_{5}^{+}	537.2490	537.2483	-1.23
b_{2}^{+}	211.1441	211.1436	-2.52
b_{3}^{+}	308.1969	308.1959	-3.07
b_{4}^{+}	379.2340	379.2331	-2.43
b_{5}^{+}	482.2432	482.2420	-2.43
b_{6}^{+}	629.3116	629.3114	-0.31
b_{7}^{+}	712.3487	712.3490	0.39
b_{8}^{+}	825.4328	825.4327	-0.05
b_{9}^{+}	882.4542	882.4529	-1.43
b_{10}^{+}	995.5383	995.5360	-2.27
b_{11}^{+}	1052.5597	1052.5572	-2.42
b_{12}^{+}	1151.6282	1151.6255	-2.27
b_{13}^{+}	1208.6496	1208.6470	-2.18

Table S9. OD_{600} of *L. lactis* sp. cremoris growing in GM17 media with different concentrations of cytolysin ($CylL_L$" + $CylL_S$" in a 1:1 ratio) for 16 h. Each experiment was performed in triplicate named 1, 2, 3. Conditional formatting color scale was applied to visualize data differences compared to the negative control (green) and the positive control (yellow).

Conc.(nM)	WT1	WT2	WT3	WT1 [c]	WT2 [c]	WT3 [c]
256	0.065	0.066	0.068	0.066	0.068	0.069
128	0.070	0.064	0.067	0.066	0.068	0.069
64	0.067	0.066	0.067	0.068	0.069	0.068
32	0.065	0.065	0.064	0.066	0.067	0.067
16	0.066	0.067	0.065	0.068	0.068	0.067
8	0.069	0.092	0.076	0.067	0.068	0.067
4	0.067	0.065	0.066	0.067	0.067	0.067
2	0.137	0.064	0.063	0.137	0.177	0.260
Control [a]	0.033			0.048		
Growth [b]	1.013			0.876		

[a] Negative control: GM17 media only. [b] Positive control: 100 μL *L. lactis* sp. cremoris culture in 100 μL GM17 media without antibiotics.
[c] Parallel experiment using another independent single-colony culture.

Table S10. OD_{600} of *L. lactis* sp. cremoris growing in GM17 media with different concentrations of dimethylated cytolysin (DiMe-$CylL_L$" + DiMe-$CylL_S$" in a 1:1 ratio) for 16 h. Each experiment was performed in triplicate named 1, 2, 3. Conditional formatting color scale was applied to visualize data differences compared to the negative control (green) and the positive control (yellow).

Conc.(nM)	DiMe1	DiMe2	DiMe3	DiMe1 [c]	DiMe2 [c]	DiMe3 [c]
256	0.070	0.072	0.090	0.069	0.072	0.072
128	0.067	0.079	0.070	0.071	0.070	0.070
64	0.069	0.073	0.074	0.068	0.069	0.070
32	0.084	0.095	0.069	0.082	0.092	0.082
16	0.725	0.751	0.719	0.795	0.782	0.767
8	0.725	0.695	0.895	0.823	0.816	0.816
4	0.775	0.839	1.092	0.859	0.851	0.849
2	0.807	0.961	1.170	0.871	0.858	0.876
Control [a]	0.033			0.048		
Growth [b]	1.013			0.876		

[a] Negative control: GM17 media only. [b] Positive control: 100 μL *L. lactis* sp. cremoris culture in 100 μL GM17 media without antibiotics.
[c] Parallel experiment using another independent single-colony culture.

Table S11. OD_{600} of *L. lactis* sp. cremoris growing in GM17 media with different concentrations of cytolysin (DiMe-CylL$_L$" + CylL$_S$" in a 1:1 ratio) for 16 h. Each experiment was performed in triplicate named 1, 2, 3. Conditional formatting color scale was applied to visualize data differences compared to the negative control (green) and the positive control (yellow).

Conc.(nM)	DiMeL+S 1	DiMeL+S 2	DiMeL+S 3	DiMeL+S 1[c]	DiMeL+S 2[c]	DiMeL+S 3[c]
256	0.072	0.071	0.071	0.077	0.095	0.069
128	0.069	0.068	0.067	0.084	0.074	0.065
64	0.077	0.076	0.072	0.068	0.073	0.069
32	0.104	0.101	0.088	0.098	0.084	0.086
16	0.769	0.786	0.734	0.738	0.761	0.673
8	0.786	0.760	0.777	0.734	0.758	0.713
4	0.798	0.792	0.796	0.760	0.772	0.728
2	0.849	0.839	0.876	0.781	0.790	0.796
Control [a]	0.050			0.048		
Growth [b]	1.125			0.714		

[a] Negative control: GM17 media only.

[b] Positive control: 100 µL *L. lactis* sp. cremoris culture in 100 µL GM17 media without antibiotics.

[c] Parallel experiment using another independent single-colony culture.

Table S12. OD_{600} of *L. lactis* sp. cremoris growing in GM17 media with different concentrations of dimethylated cytolysin (CylL$_L$" + DiMe-CylL$_S$" in a 1:1 ratio) for 16 h. Each experiment was performed in triplicate named 1, 2, 3. Conditional formatting color scale was applied to visualize data differences compared to the negative control (green) and the positive control (yellow).

Conc.(nM)	L+DiMeS 1	L+DiMeS 2	L+DiMeS 3	L+DiMeS 1[c]	L+DiMeS 2[c]	L+DiMeS 3[c]
256	0.069	0.0762	0.0678	0.0686	0.0675	0.0692
128	0.0672	0.0657	0.0654	0.0687	0.0721	0.0652
64	0.0731	0.0682	0.0779	0.0833	0.0785	0.0693
32	0.0742	0.0657	0.0655	0.0665	0.0735	0.0675
16	0.0682	0.0679	0.076	0.0691	0.0673	0.0675
8	0.0718	0.0659	0.0698	0.0663	0.0663	0.0718
4	0.0889	0.0726	0.0858	0.1634	0.2171	0.2291
2	0.8443	0.7645	0.8314	0.7277	0.7646	0.7416
Control [a]	0.0501			0.0483		
Growth [b]	1.1252			0.7141		

[a] Negative control: GM17 media only.

[b] Positive control: 100 µL *L. lactis* sp. cremoris culture in 100 µL GM17 media without antibiotics.

[c] Parallel experiment using another independent single-colony culture.

Table S13. OD_{600} of *L. lactis* sp. cremoris growing in GM17 media with different concentrations of haloduracin (Halα + Halβ in a 1:1 ratio) for 16 h. Each experiment was performed in triplicate named 1, 2, 3. Conditional formatting color scale was applied to visualize data differences compared to the negative control (green) and the positive control (yellow).

Conc. (nM)	WT1	WT2	WT3	WT1[c]	WT2[c]	WT3[c]
256	0.097	0.097	0.098	0.101	0.097	0.098
128	0.098	0.101	0.100	0.098	0.101	0.100
64	0.102	0.107	0.114	0.102	0.105	0.104
32	0.113	0.103	0.111	0.118	0.107	0.124
16	0.147	0.144	0.209	0.175	0.186	0.282
8	1.039	1.097	1.110	0.982	1.015	1.041
4	1.061	1.122	1.078	1.033	1.039	1.004
2	1.043	1.083	1.079	1.050	1.040	1.048
Control [a]	0.059			0.058		
Growth [b]	1.268			1.137		

[a] Negative control: GM17 media only.

[b] Positive control: 100 μL *L. lactis* sp. cremoris culture in 100 μL GM17 media without antibiotics.

[c] Parallel experiment using another independent single-colony culture.

Table S14. OD_{600} of *L. lactis* sp. cremoris growing in GM17 media with different concentrations of dimethylated haloduracin β (Halα + DiMe-Halβ in a 1:1 ratio) for 16 h. Each experiment was performed in triplicate named 1, 2, 3. Conditional formatting color scale was applied to visualize data differences compared to the negative control (green) and the positive control (yellow).

Conc. (nM)	α+DiMeβ 1	α+DiMeβ 2	α+DiMeβ 3	α+DiMeβ 1[c]	α+DiMeβ 2[c]	α+DiMeβ 3[c]
256	0.102	0.100	0.098	0.110	0.100	0.098
128	0.103	0.104	0.101	0.103	0.104	0.102
64	0.102	0.118	0.102	0.102	0.120	0.102
32	0.103	0.103	0.107	0.103	0.103	0.111
16	0.106	0.107	0.106	0.105	0.107	0.107
8	0.110	0.185	0.108	0.111	0.273	0.110
4	1.072	1.070	1.014	0.973	0.988	1.030
2	1.117	1.094	1.075	1.057	1.04	1.071
Control [a]	0.059			0.058		
Growth [b]	1.268			1.137		

[a] Negative control: GM17 media only.

[b] Positive control: 100 μL *L. lactis* sp. cremoris single-culture in 100 μL GM17 media without antibiotics.

[c] Parallel experiment using another independent colony culture.

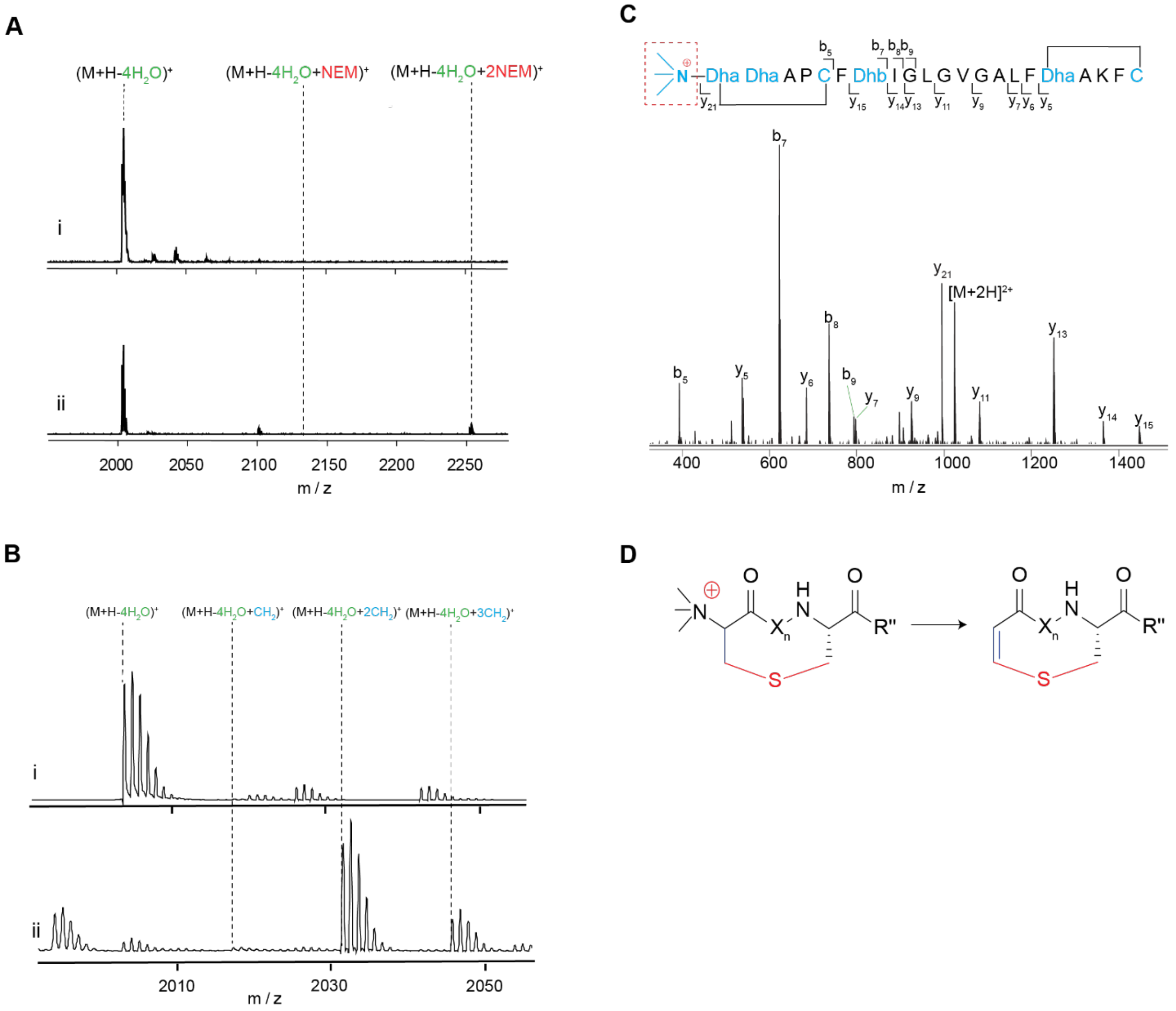

Figure S1. (A) MALDI-TOF MS of the CylM-modified, CylA-digested His$_6$–CylL$_S$-(T1S/T2S/P3A/A4P) before NEM treatment (i) and after NEM treatment (ii). In (i), $(M + H – 4\ H_2O)^+$ calcd. *m/z* = 2004.0, obsd. *m/z*= 2003.7. In (ii), $(M + H – 4\ H_2O)^+$ calcd. *m/z* = 2004.0, obsd. *m/z*= 2004.0; $(M + H – 4\ H_2O + NEM)^+$ calcd. *m/z* = 2129.1; $(M + H – 4\ H_2O + 2\ NEM)^+$ calcd. *m/z* = 2254.2. **(B)** MALDI-TOF MS of CylL$_S$-(T1S/T2S/P3A/A4P) before CaoS$_C$ treatment (i) and after 24 h CaoS$_C$ treatment (ii). In (i), $(M + H – 4\ H_2O)^+$ calcd. *m/z* = 2004.0, obsd. *m/z*= 2004.1. In (ii), $(M + H – 4\ H_2O + CH_2)^+$ calcd. *m/z* = 2018.0; $(M + H – 4\ H_2O + 2\ CH_2)^+$ calcd. *m/z* = 2032.0, obsd. *m/z*= 2032.1; $(M + H – 4\ H_2O + 3\ CH_2)^+$ calcd. *m/z* = 2046.0, obsd. *m/z*= 2046.1. **(C)** LC-ESI-QTOF MS–MS fragmentation pattern of the trimethylated CylL$_S$-(T1S/T2S/P3A/A4P) catalyzed by CaoS$_C$. For fragment masses, see Table S3. **(D)** Deamination reaction on the N-terminus of trimethylated CylL$_S$-(T1S/T2S/P3A/A4P) in LC-ESI-QTOF.[3]

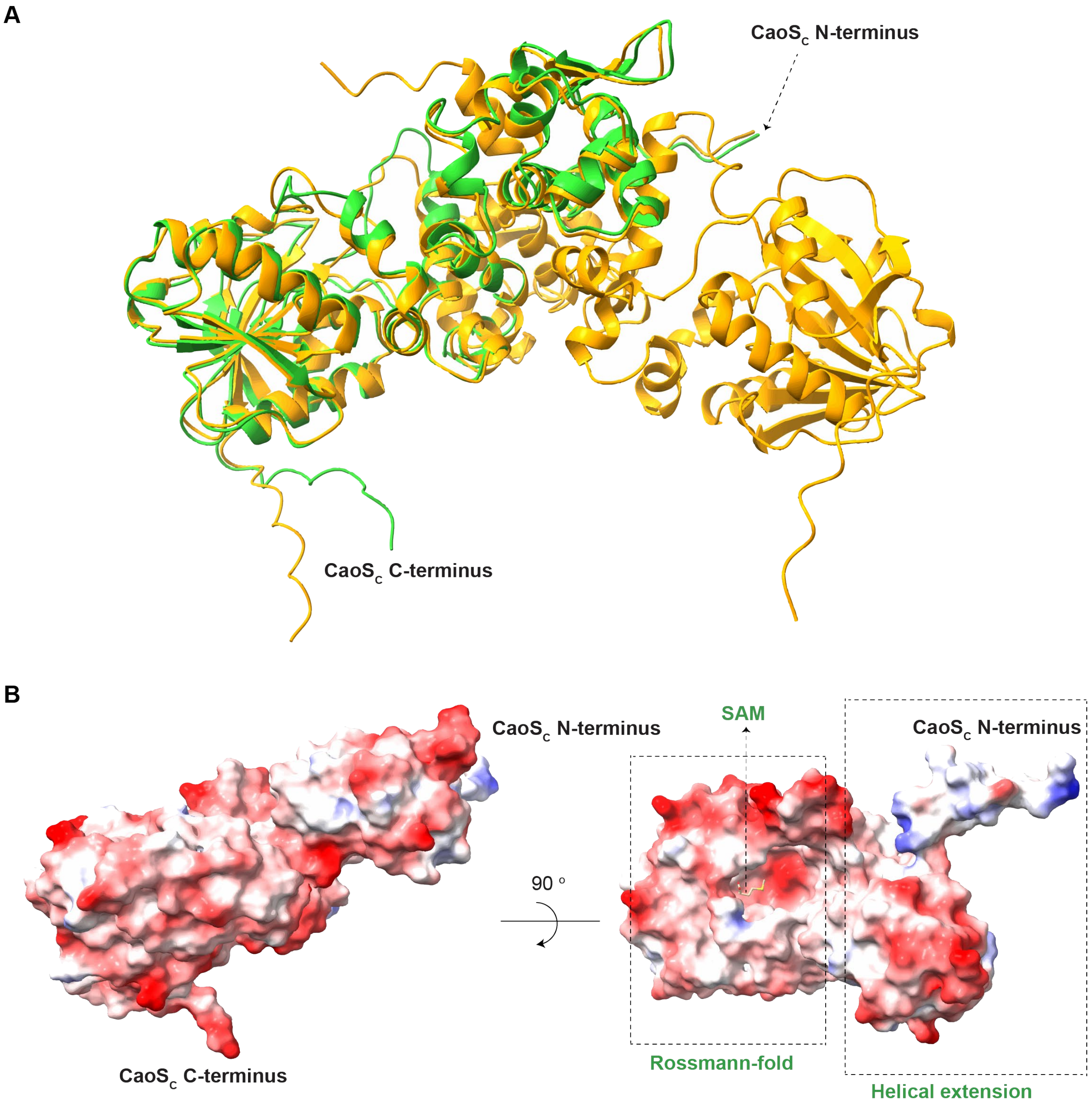

Figure S2. (A) $CaoS_C$ homodimer (orange) aligned with rank_1 model of the $CaoS_C$ monomer (green) (RMSD of 1.1 Å over 2,301 aligned Cα atoms). **(B)** $CaoS_C$ monomer is colored in surface rendering based on electrostatics, with negatively charged surface in red, and positively charged surface in navy.

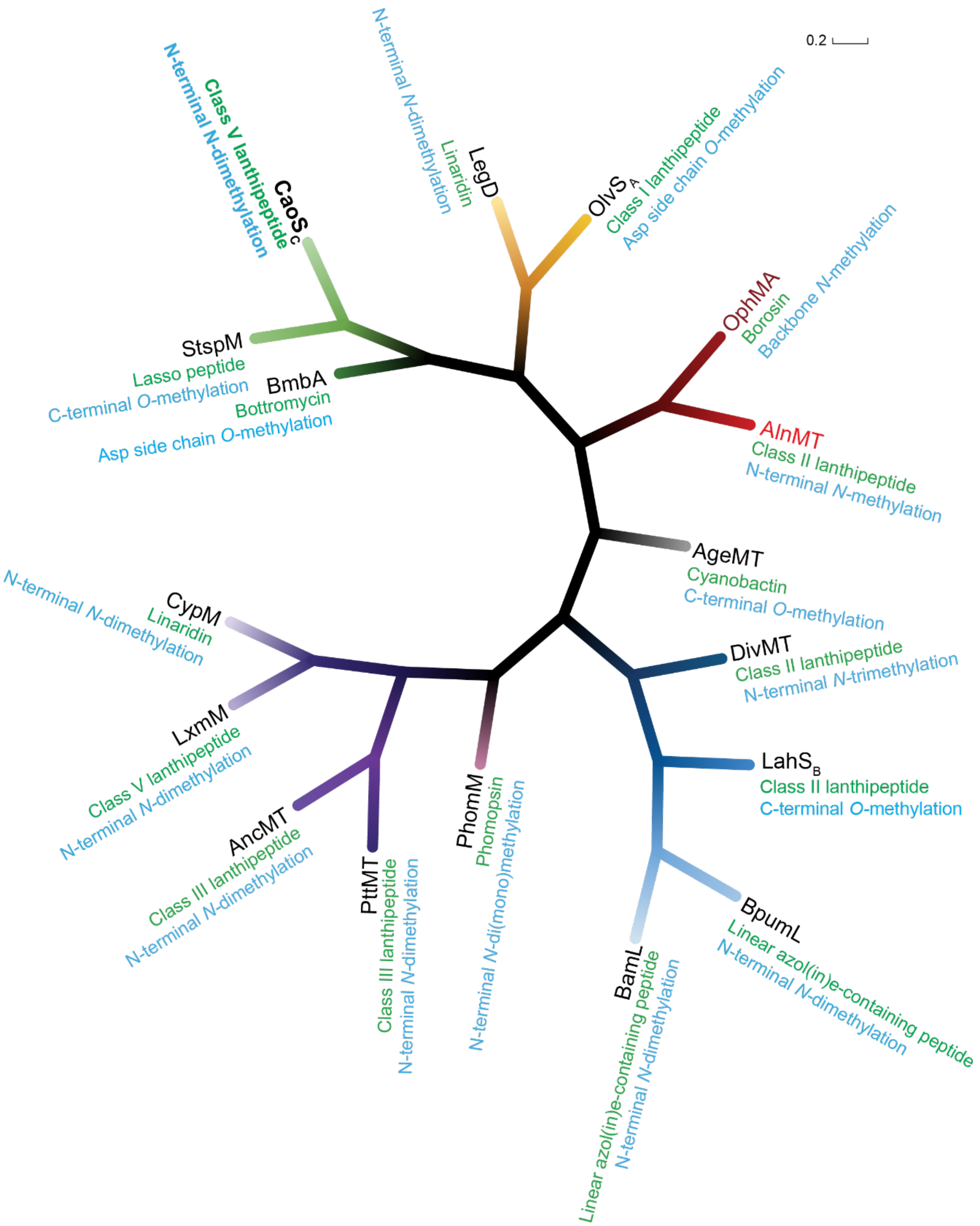

Figure S3. Phylogenetic tree of characterized methyltransferases involved in the biosynthesis of RiPPs.[4] In the analysis, 17 enzymes were used that catalyze methylation of members of various RiPP classes indicated in green. The catalytic functions are in blue. Class I methyltransferases are colored in black with CaoS$_C$ bolded, class III methyltransferase (OphMA) is in dark red, and the putative radical SAM methyltransferase (AlnMT) is in red. This unrooted tree was generated through EMBL-EBI multiple sequence alignment (MSA) of the 17 enzymes, followed by iTOL phylogenetic tree analysis.[5]

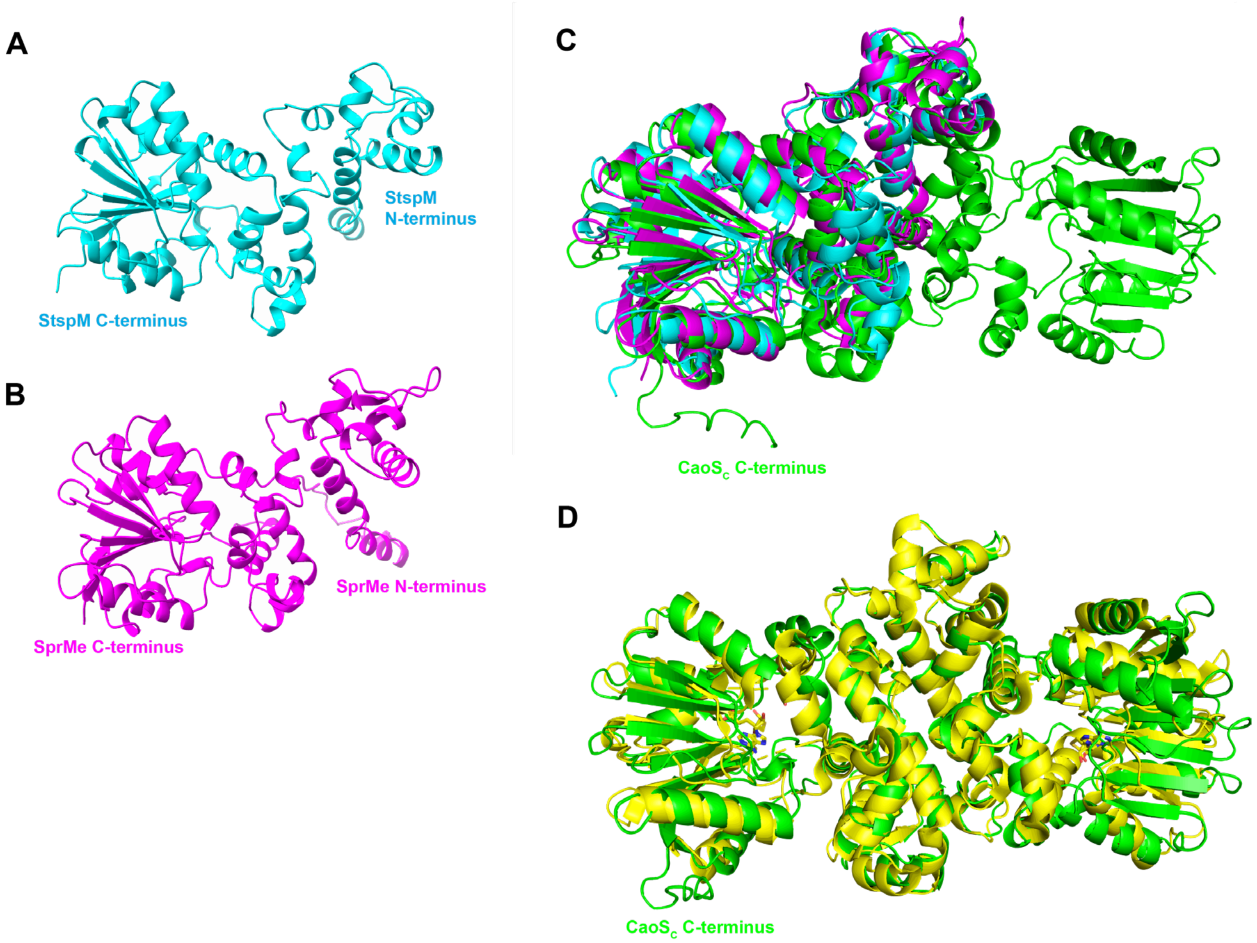

Figure S4. Structure of the NcsB1 family methyltransferases involved in RiPP biosynthesis, including AlphaFold2-predicted models of two *N*-methyltransferases **(A)** StspM and **(B)** SprMe. Previous studies reported that StspM is a monomer,[6] whereas NcsB1 is a dimer.[7] **(C)** Alignment of the predicted structures of StspM and SprMe with the CaoS$_C$ homodimer (CaoS$_C$ with StspM, RMSD of 3.8 Å over 1,778 atoms; with SprMe, RMSD of 2.5 Å over 2,040 atoms). (**D**) Alignment of the *O*-methyltransferase NcsB1 crystal structure (homodimer in yellow, PDB ID: 3I53) with the CaoS$_C$ predicted homodimer structure (RMSD of 3.7 Å over 3,348 atoms).

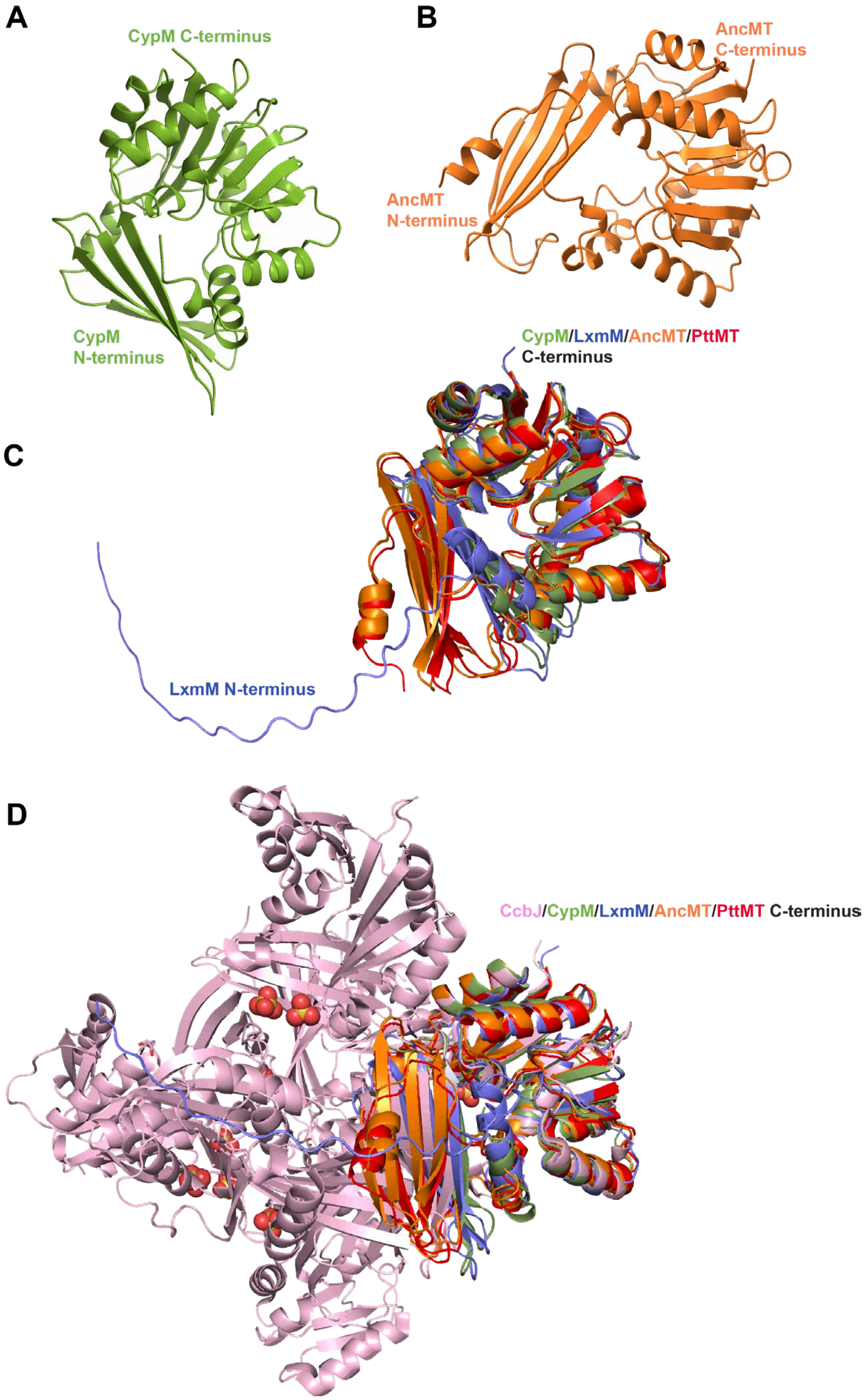

Figure S5. Structures of CcbJ family *N*-methyltransferases involved in RiPP biosynthesis, including predicted models of **(A)** CypM, **(B)** AncMT, and **(C)** their aligned structures with LxmM and PttMT (PttMT with CypM, RMSD of 2.0 Å over 1,078 atoms; with AncMT, RMSD of 0.3 Å over 1,644 atoms; with LxmM, RMSD of 2.0 Å over 1,045 atoms). (**D**) Structural alignment of the CcbJ crystal structure (homohexamer in pink, PDB ID: 4HGY) with CypM, LxmM, AncMT and PttMT. PttMT with CcbJ, RMSD of 1.8 Å over 1,008 atoms.

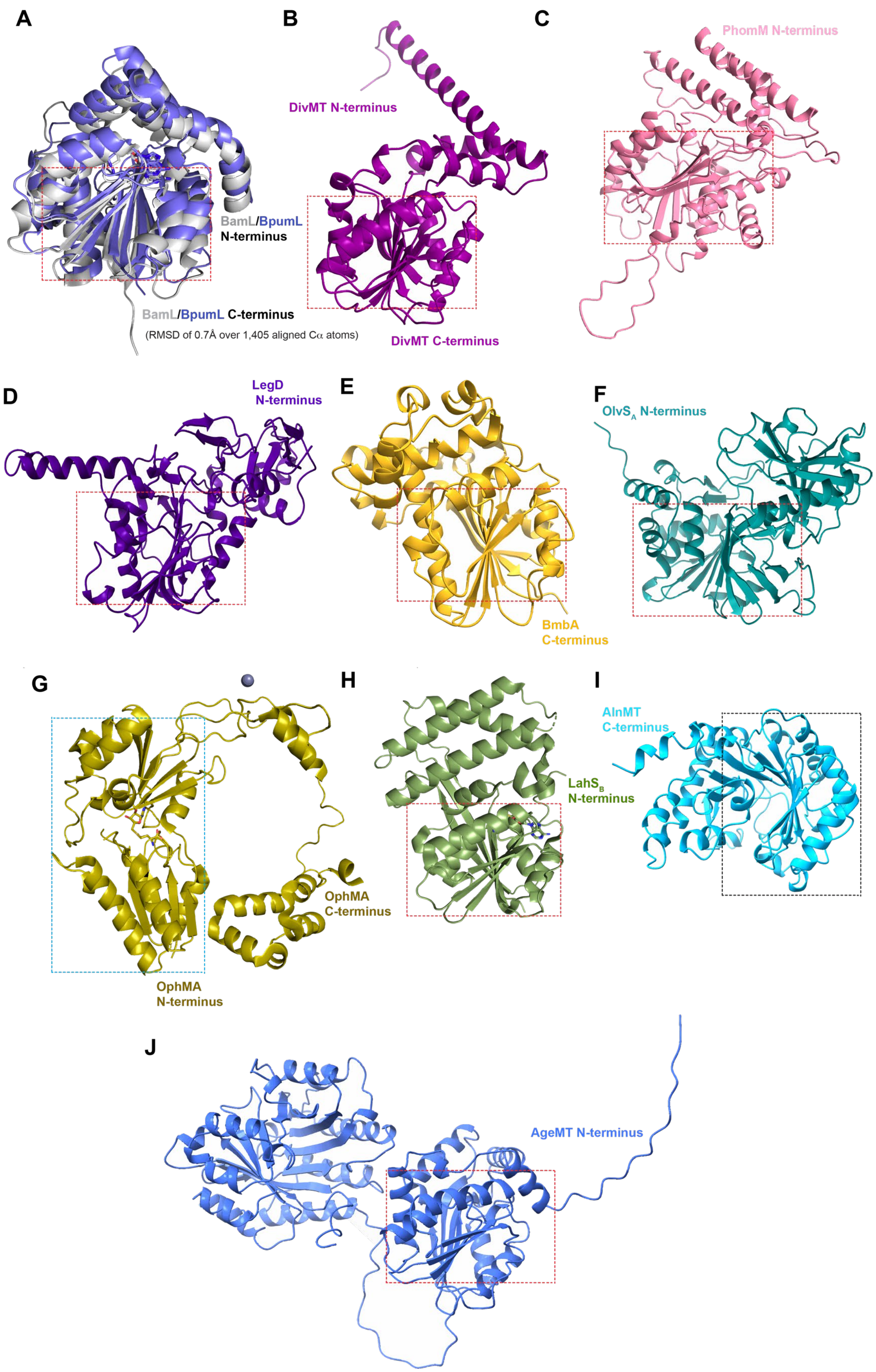
A
B
C
PhomM N-terminus
DivMT N-terminus
BamL/BpumL
N-terminus
BamL/BpumL C-terminus
(RMSD of 0.7Å over 1,405 aligned Cα atoms)
DivMT C-terminus
D
LegD
N-terminus
E
F
OlvS$_A$ N-terminus
BmbA
C-terminus
G
H
I
AlnMT
C-terminus
LahS$_B$
N-terminus
OphMA
C-terminus
OphMA
N-terminus
J
AgeMT N-terminus

Figure S6. Structures of characterized and proposed methyltransferases in RiPP biosynthesis, including crystal structures of BamL (PDB ID: 4KVZ)/BpumL (PDB ID: 4KWC) **(A)**, OphMA (PDB ID: 6TSC) **(G)**, and LahS$_B$ (PDB ID: 6UAK) **(H)**, and predicted models of DivMT **(B)**, PhomM **(C)**, LegD **(D)**, BmbA (**E**), OlvS$_A$ **(F)**, AlnMT **(I)** and AgeMT **(J)**. The class I methyltransferase Rossmann-fold is framed in red boxes, the class III methyltransferase SAM binding site is framed in blue, and the radical SAM domain is framed in black.

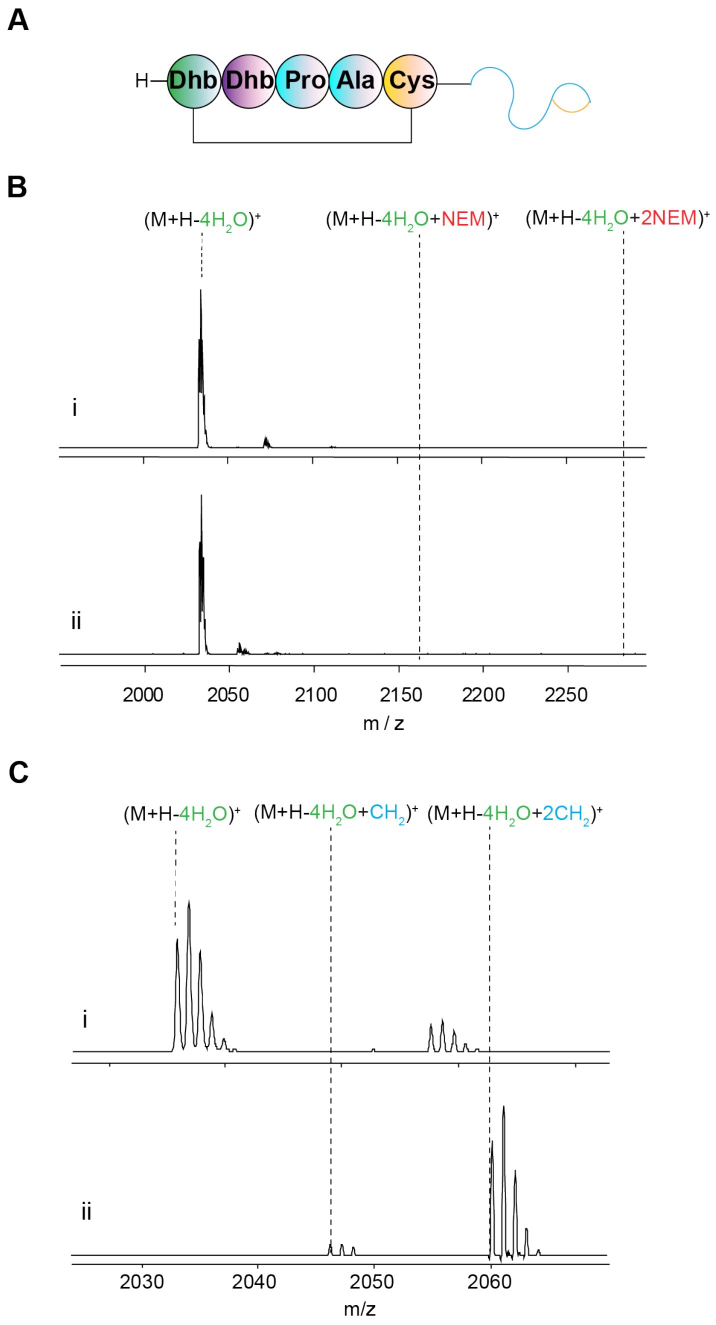

Figure S7. **(A)** Schematic representation of CylM-modified, CylA-digested His$_6$–CylL$_S$. The N-terminal sequence of the peptide is described in detail, with a short-hand depiction of the C-terminal structure. **(B)** MALDI-TOF MS of CylL$_S$" before NEM treatment (i) and after NEM treatment (ii). In (i), $(M + H – 4\ H_2O)^+$ calcd. *m/z* = 2032.0, obsd. *m/z*= 2031.3. In (ii), $(M + H – 4\ H_2O)^+$ calcd. *m/z* = 2032.0, obsd. *m/z*= 2031.9; $(M + H – 4\ H_2O + NEM)^+$ calcd. *m/z* = 2157.2; $(M + H – 4\ H_2O+ 2\ NEM)^+$ calcd. *m/z* = 2282.3. **(C)** MALDI-TOF MS of CylL$_S$" before CaoS$_C$ treatment (i) and after CaoS$_C$ treatment (ii). In (i), $(M + H – 4\ H_2O)^+$ calcd. *m/z* = 2032.0. In (ii), $(M + H – 4\ H_2O + CH_2)^+$ calcd. *m/z* = 2046.0, obsd. *m/z*= 2046.1; $(M + H – 4\ H_2O+ 2\ CH_2)^+$ calcd. *m/z* = 2060.0, obsd. *m/z*= 2060.1.

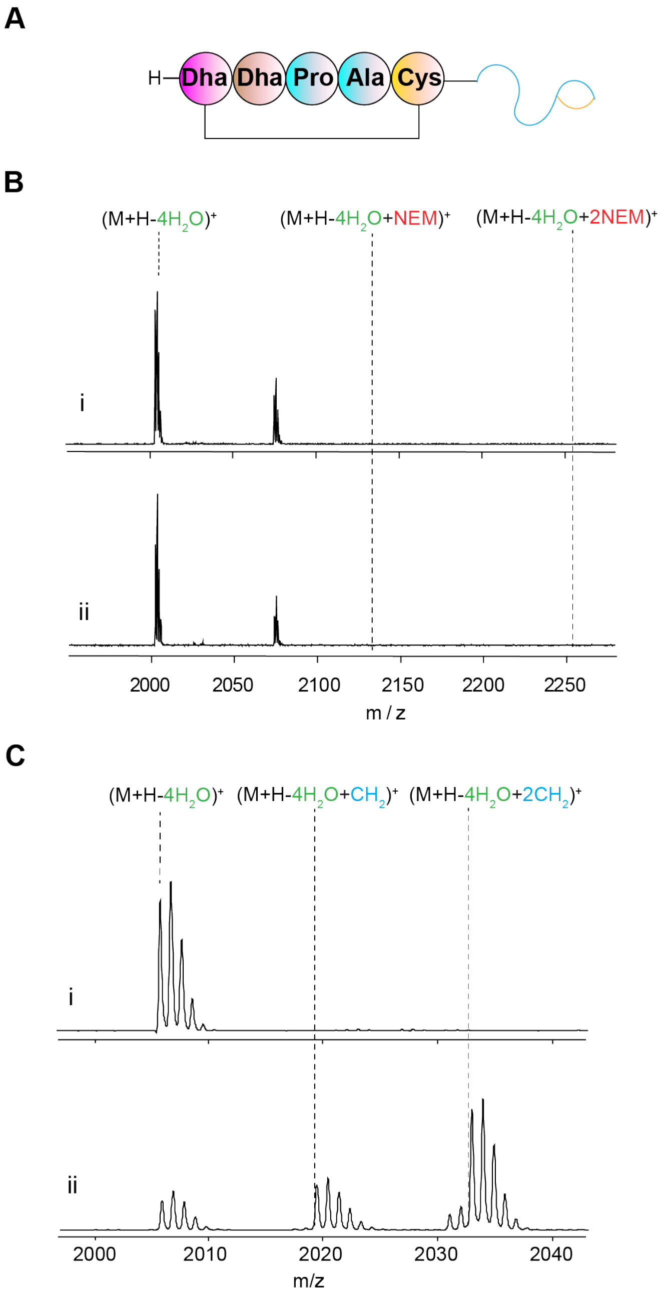

Figure S8. (A) Schematic representation of CylM-modified, CylA-digested His_6–$CylL_S$-(T1S/T2S). The N-terminal sequence of the peptide is described in detail, with a short-hand depiction of the C-terminal structure. **(B)** MALDI-TOF MS of the peptide described in (A) before NEM treatment (i) and after NEM treatment (ii). In (i), $(M + H – 4\ H_2O)^+$ calcd. *m/z* = 2004.0, obsd. *m/z*= 2004.0. In (ii), $(M + H – 4\ H_2O)^+$ calcd. *m/z* = 2004.0, obsd. *m/z*= 2004.4; $(M + H – 4\ H_2O + NEM)^+$ calcd. *m/z* = 2129.1; $(M + H – 4\ H_2O+ 2\ NEM)^+$ calcd. *m/z* = 2254.2. **(C)** MALDI-TOF MS of $CylL_S$-(T1S/T2S) before $CaoS_C$ treatment (i) and after $CaoS_C$ treatment (ii). In (i), $(M + H – 4\ H_2O)^+$ calcd. *m/z* = 2004.0, obsd. *m/z*= 2004.4. In (ii), $(M + H – 4\ H_2O)^+$ calcd. *m/z* =

2004.0, obsd. *m/z*= 2003.2; $(M + H - 4\ H_2O + CH_2)^+$ calcd. *m/z* = 2018.0, obsd. *m/z*= 2017.2; $(M + H - 4\ H_2O + 2\ CH_2)^+$ calcd. *m/z* = 2032.0, obsd. *m/z*= 2031.3.

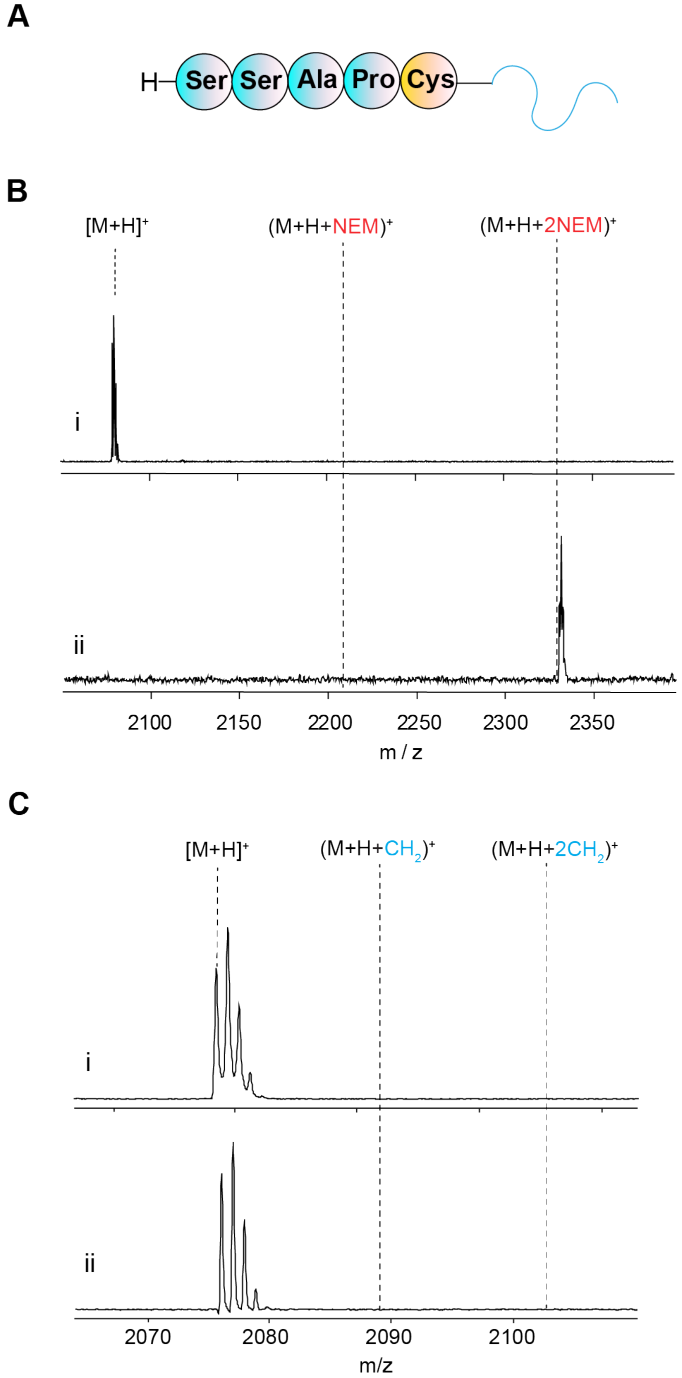

Figure S9. (A) Schematic representation of CylA-digested unmodified His_6–$CylL_S$-(T1S/T2S/P3A/A4P). The N-terminal sequence of the peptide is described in detail, with a short-hand depiction of the C-terminal structure. **(B)** MALDI-TOF MS of the peptide described in (A) before NEM treatment (i) and after NEM treatment (ii). In (i), $[M + H]^+$ calcd. *m/z* = 2076.0, obsd. *m/z*= 2075.9. In (ii), $[M + H]^+$ calcd. *m/z* = 2076.0; $(M + H + NEM)^+$ calcd. *m/z* = 2201.2; $(M + H + 2\ NEM)^+$ calcd. *m/z* =2326.3, obsd. *m/z*= 2326.5. **(C)** MALDI-TOF MS of $CylL_S$-(T1S/T2S/P3A/A4P) before $CaoS_C$ treatment (i) and after $CaoS_C$ treatment (ii). In (i), $[M + H]^+$

calcd. *m/z* = 2076.0, obsd. *m/z*= 2075.9. In (ii), $[M + H]^+$ calcd. *m/z* = 2076.0, obsd. *m/z*= 2075.7; $(M + H + CH_2)^+$ calcd. *m/z* = 2090.0; $(M + H + 2\ CH_2)^+$ calcd. *m/z* =2104.0.

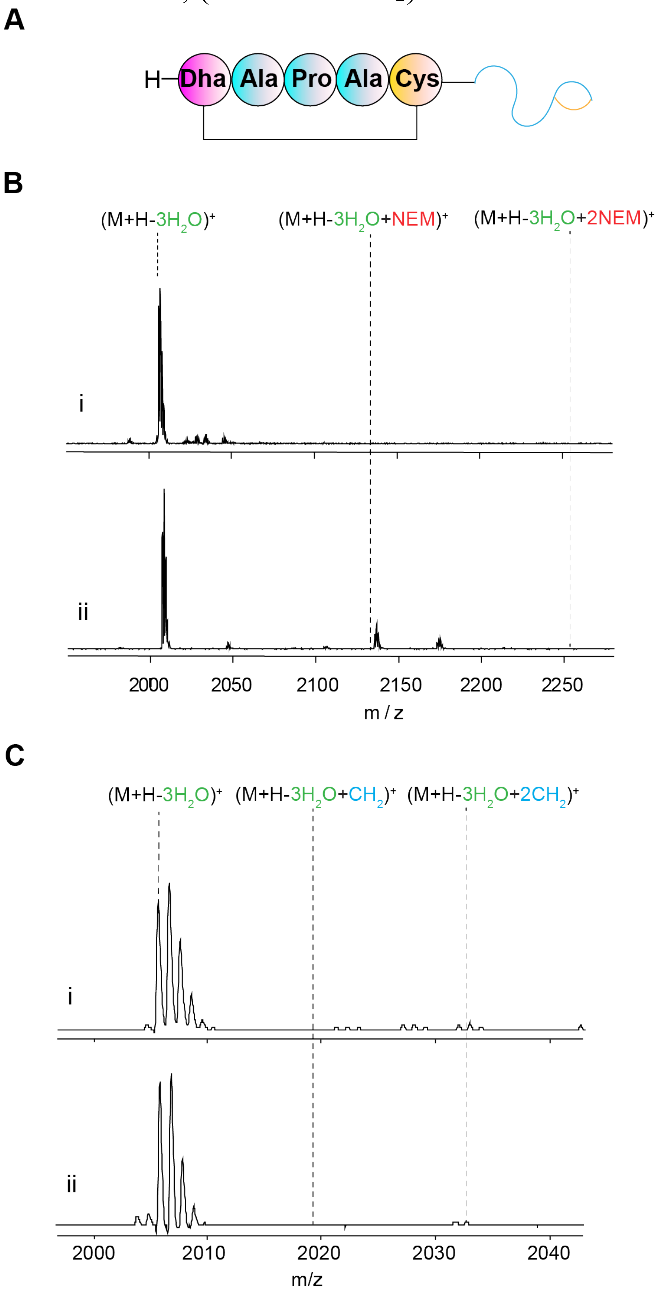

Figure S10. (A) Schematic representation of CylM-modified, CylA-digested His_6–$CylL_S$-(T1S/T2A). The N-terminal sequence of the peptide is described in detail, with a short-hand depiction of the C-terminal structure. **(B)** MALDI-TOF MS of the peptide described in (A) before NEM treatment (i) and after NEM treatment (ii). In (i), $(M + H – 3\ H_2O)^+$ calcd. *m/z* = 2006.0, obsd. *m/z*= 2005.4. In (ii), $(M + H – 3\ H_2O)^+$ calcd. *m/z* = 2006.0, obsd. *m/z*= 2006.4; $(M + H – 3\ H_2O + NEM)^+$ calcd. *m/z* = 2131.1; $(M + H – 3\ H_2O+ 2\ NEM)^+$ calcd. *m/z* = 2256.3. **(C)** MALDI-TOF MS of $CylL_S$-(T1S/T2A) before $CaoS_C$ treatment (i) and after $CaoS_C$ treatment (ii). In (i), $(M + H – 3\ H_2O)^+$ calcd. *m/z* = 2006.0, obsd. *m/z*= 2005.4; In (ii), $(M + H – 3\ H_2O)^+$ calcd. *m/z* =

2006.0, obsd. *m/z*= 2006.1; $(M + H - 3\ H_2O + CH_2)^+$ calcd. *m/z* = 2020.0; $(M + H - 3\ H_2O + 2\ CH_2)^+$ calcd. *m/z* = 2034.0.

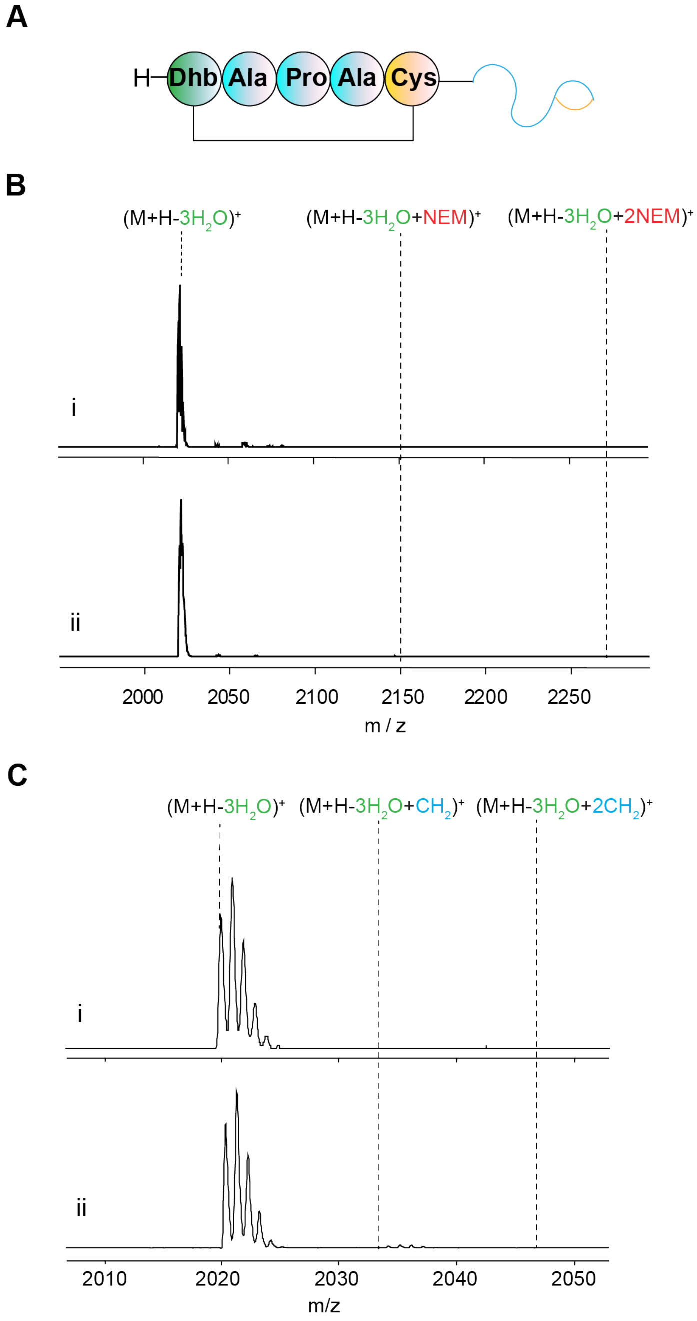

Figure S11. (A) Schematic representation of CylM-modified, CylA-digested His_6–$CylL_S$-(T2A). The N-terminal sequence of the peptide is described in detail, with a short-hand depiction of the C-terminal structure. **(B)** MALDI-TOF MS of the peptide described in (A) before NEM treatment (i) and after NEM treatment (ii). In (i), $(M + H - 3\ H_2O)^+$ calcd. *m/z* = 2020.0, obsd. *m/z*= 2019.9. In (ii), $(M + H - 3\ H_2O)^+$ calcd. *m/z* = 2020.0, obsd. *m/z*= 2020.0; $(M + H - 3\ H_2O + NEM)^+$ calcd. *m/z* = 2145.2; $(M + H - 3\ H_2O + 2\ NEM)^+$ calcd. *m/z* = 2270.3. **(C)** MALDI-TOF MS of $CylL_S$-(T2A) before $CaoS_C$ treatment (i) and after $CaoS_C$ treatment (ii). In (i), $(M + H - 3\ H_2O)^+$ calcd.

m/z = 2020.0, obsd. *m/z*= 2019.9; In (ii), $(M + H - 3\ H_2O)^+$ calcd. *m/z* = 2020.0, obsd. *m/z*= 2020.0; $(M + H - 3\ H_2O + CH_2)^+$ calcd. *m/z* = 2034.0; $(M + H - 3\ H_2O + 2\ CH_2)^+$ calcd. *m/z* = 2048.0.

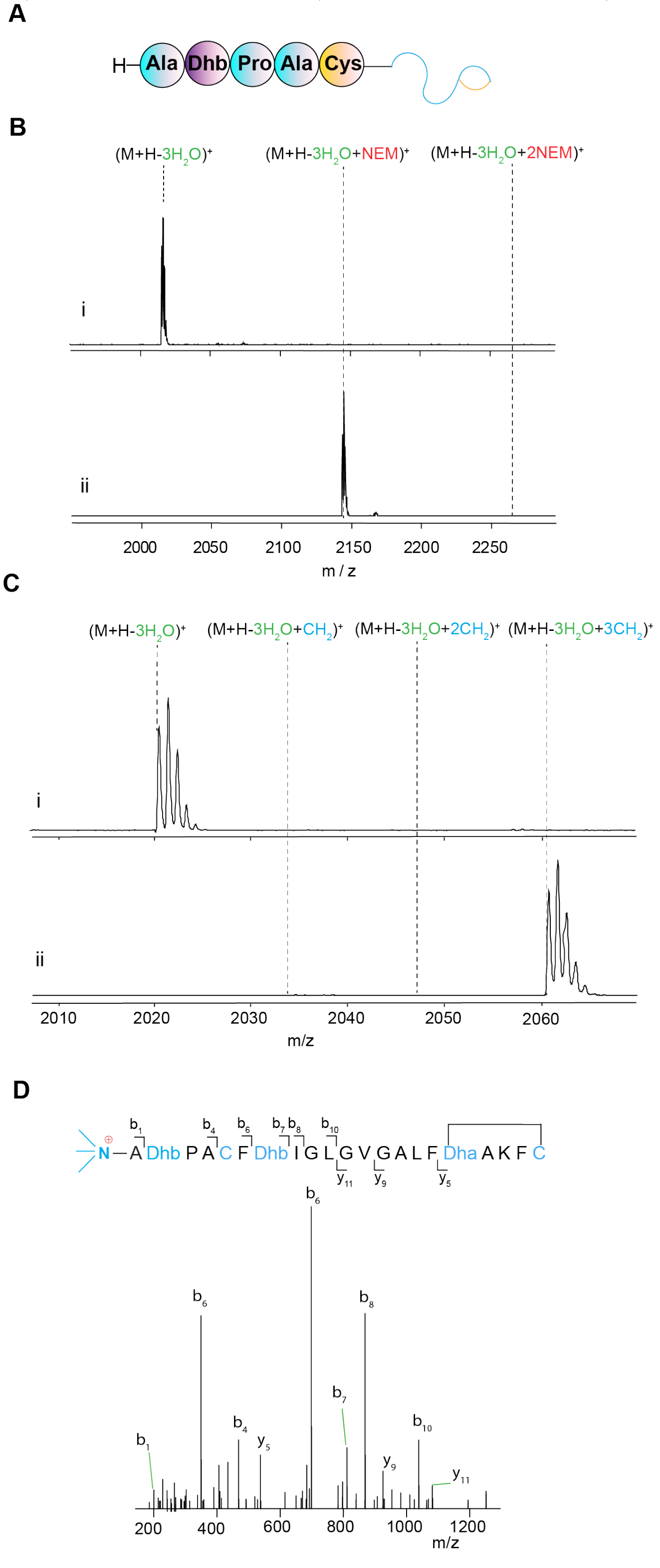

Figure S12. (A) Schematic representation of CylM-modified, CylA-digested His$_6$–CylL$_S$-(T1A). The N-terminal sequence of the peptide is described in detail, with a short-hand depiction of the C-terminal structure. **(B)** MALDI-TOF MS of the peptide described in (A) before NEM treatment (i) and after NEM treatment (ii). In (i), $(M + H - 3\ H_2O)^+$ calcd. $m/z = 2020.0$, obsd. $m/z = 2020.1$. In (ii) $(M + H - 3\ H_2O + NEM)^+$ calcd. $m/z = 2145.2$, obsd. $m/z = 2145.0$; $(M + H - 3\ H_2O + 2\ NEM)^+$ calcd. $m/z = 2270.3$. **(C)** MALDI-TOF MS of CylL$_S$-(T1A) before CaoS$_C$ treatment (i) and after CaoS$_C$ treatment (ii). In (i), $(M + H - 3\ H_2O)^+$ calcd. $m/z = 2020.0$, obsd. $m/z = 2020.1$; In (ii), $(M + H - 3\ H_2O)^+$ calcd. $m/z = 2020.0$; $(M + H - 3\ H_2O + CH_2)^+$ calcd. $m/z = 2034.0$; $(M + H - 3\ H_2O + 2\ CH_2)^+$ calcd. $m/z = 2048.0$; $(M + H - 3\ H_2O + 3\ CH_2)^+$ calcd. $m/z = 2062.1$, obsd. $m/z = 2061.3$. **(D)** LC-ESI-QTOF MS–MS fragmentation pattern of the trimethylated CylL$_S$-(T1A) catalyzed by CaoS$_C$. For fragment masses, see Table S4.

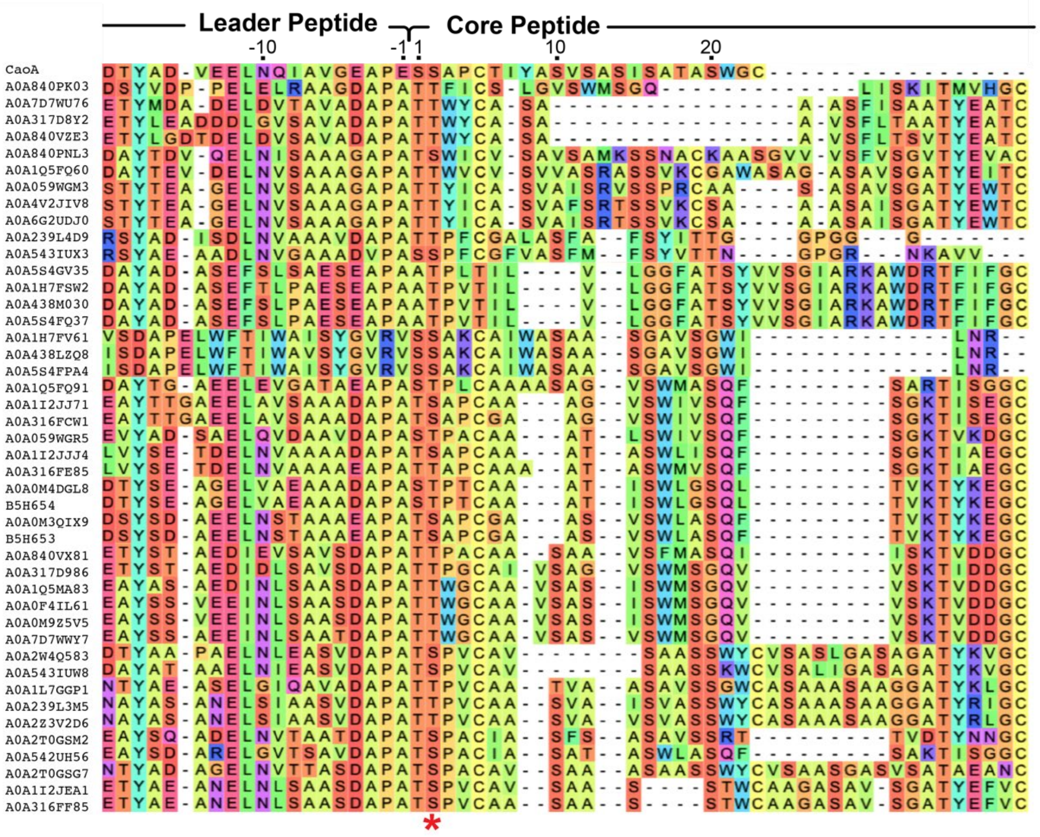

Figure S13. MSA of partial sequences of selected putative class V LanA precursor peptides that co-occur with LanKYS$_C$. The sequences were retrieved from a Pfam-based genome neighborhood network of CaoY homologues (PF17914) generated by the Enzyme Function Initiative-Enzyme Similarity Tool.[8-10] The predicted N-terminal CP sequences and C-terminal LP sequences are aligned and positioned based on CaoA residue number.[10] Each amino acid is colored differently to reflect its conservation within the MSA. Fully conserved Dhx at the second position of the core peptide is highlighted by a red asterisk. The UniProtKB ID of each peptide is depicted in the left column.

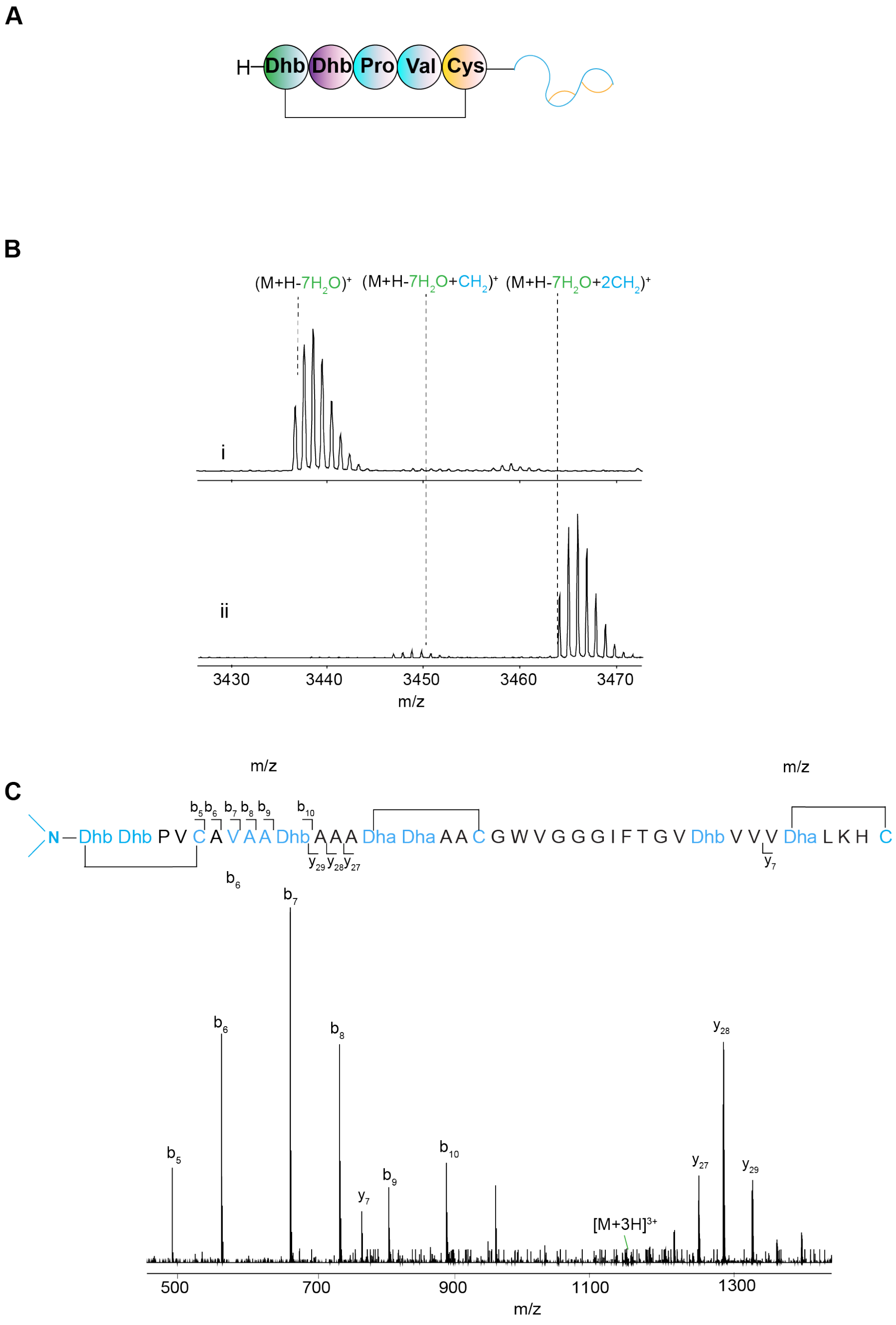

Figure S14. (A) Schematic representation of CylM-modified, CylA-digested His$_6$–CylL$_L$. The N-terminal sequence of the peptide is described in detail, with a short-hand depiction of the C-terminal structure (see Fig. 6, main text for full structure). **(B)** MALDI-TOF MS of CylL$_L$'' before CaoS$_C$ treatment (i) and after CaoS$_C$ treatment (ii). In (i), $(M + H - 7\ H_2O)^+$ calcd. *m/z* = 3436.7, obsd. *m/z*= 3436.5. In (ii), $(M + H - 7\ H_2O + CH_2)^+$ calcd. *m/z* = 3450.7; $(M + H - 7\ H_2O + 2\ CH_2)^+$ calcd. *m/z* = 3464.7, obsd. *m/z*= 3464.5. **(C)** LC-ESI-QTOF MS–MS fragmentation pattern of the dimethylated CylL$_L$'' catalyzed by CaoS$_C$. For fragment masses, see Table S5.

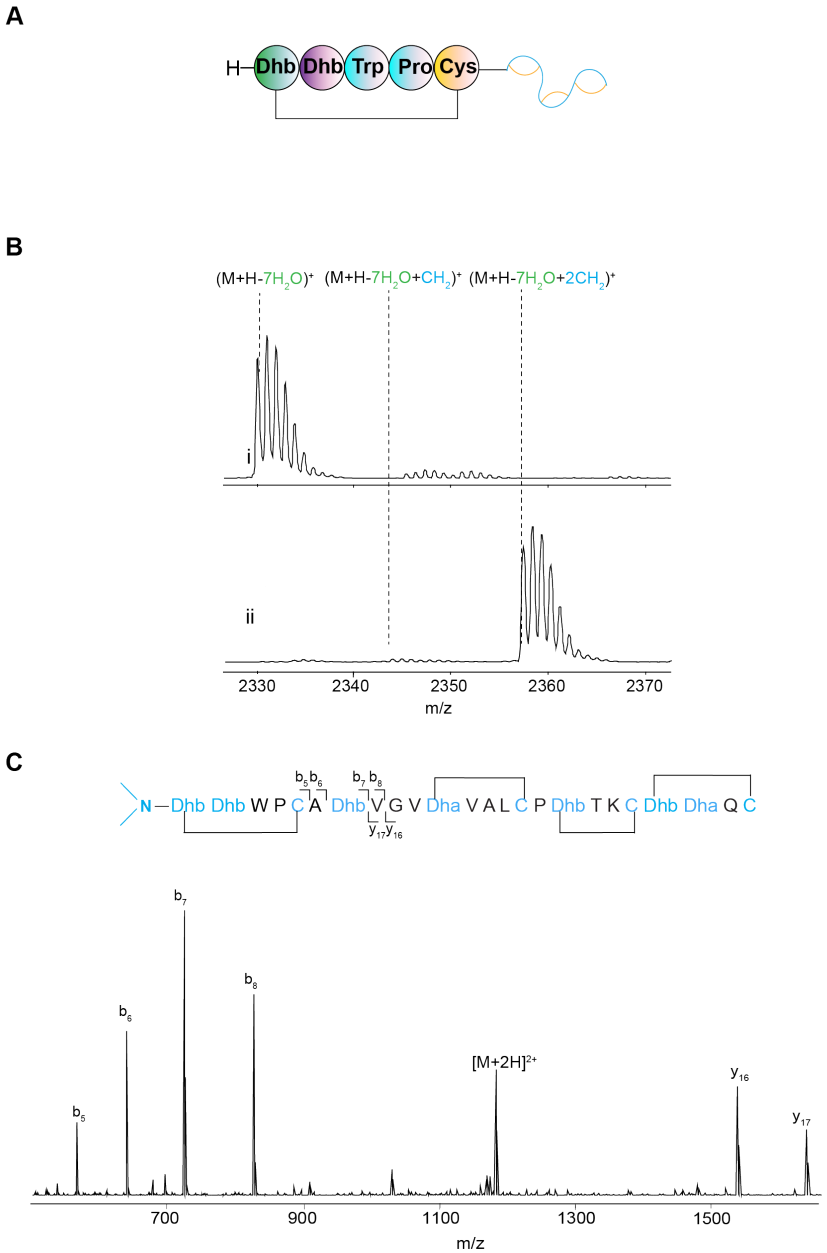

Figure S15. (A) Schematic representation of HalM2-modified, Factor Xa-digested His$_6$–HalA2.[11] The N-terminal sequence of the peptide is described in detail, with a short-hand depiction of the C-terminal structure (see Fig. 6 in main text for full structure). **(B)** MALDI-TOF MS of Halβ before CaoS$_C$ treatment (i) and after CaoS$_C$ treatment (ii). In (i), $(M + H - 7\ H_2O)^+$ calcd. *m/z* = 2331.1, obsd. *m/z*= 2330.1. In (ii), $(M + H - 7\ H_2O + CH_2)^+$ calcd. *m/z* = 2345.1; $(M + H - 7\ H_2O + 2\ CH_2)^+$ calcd. *m/z* = 2359.1, obsd. *m/z*= 2359.1. **(C)** LC-ESI-QTOF MS−MS fragmentation pattern of the dimethylated Halβ catalyzed by CaoS$_C$. For fragment masses, see Table S6.

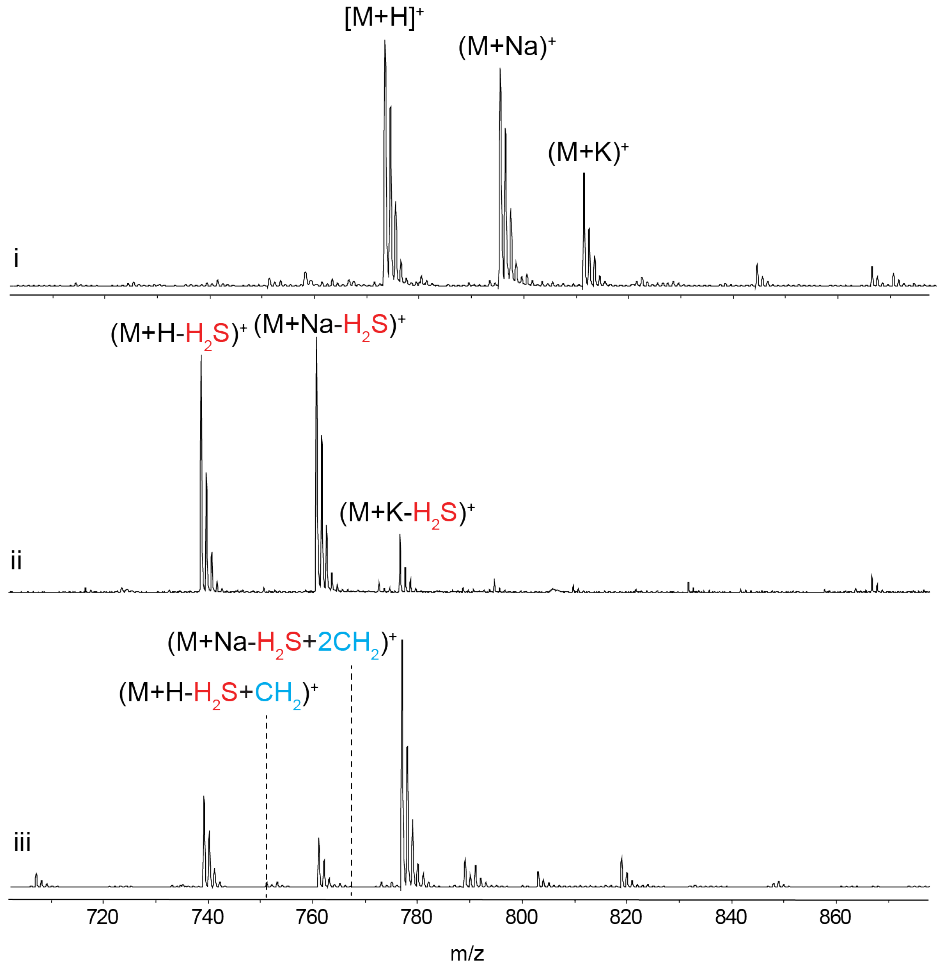

Figure S16. MALDI-TOF MS of (i) SPPS synthesized 8-residue peptide Ala1-Cys2-Ala3-Pro4-Ala5-Thr6-Ile7-Lys8. $[M + H]^+$ calcd. *m/z* = 774.4, obsd. *m/z*= 773.5; $(M + Na)^+$ calcd. *m/z* = 795.4, obsd. *m/z*= 795.5; $(M + K)^+$ calcd. *m/z* = 812.4, obsd. *m/z*= 811.5. (ii) Ala1-Dha2-Ala3-Pro4-Ala5-Thr6-Ile7-Lys8 resulted from Davis reagent[12] treatment of Ala1-Cys2-Ala3-Pro4-Ala5-Thr6-Ile7-Lys8 that leads to net H_2S elimination from Cys. $(M + H – H_2S)^+$ calcd. *m/z* = 740.4, obsd. *m/z*= 739.5; $(M + Na – H_2S)^+$ calcd. *m/z* = 762.4, obsd. *m/z*= 761.5; $(M + K – H_2S)^+$ calcd. *m/z* = 778.4, obsd. *m/z*= 777.5. (iii) Ala1-Dha2-Ala3-Pro4-Ala5-Thr6-Ile7-Lys8 after $CaoS_C$ treatment. $(M + H – H_2S)^+$ calcd. *m/z* = 740.4, obsd. *m/z*= 740.0; $(M + Na – H_2S)^+$ calcd. *m/z* = 762.4, obsd. *m/z*= 761.9; $(M + K – H_2S)^+$ calcd. *m/z* = 778.4, obsd. *m/z*= 777.9. $(M + H – H_2S + CH_2)^+$ calcd. *m/z* = 754.4; $(M + H – H_2S + 2\ CH_2)^+$ calcd. *m/z* =768.4.

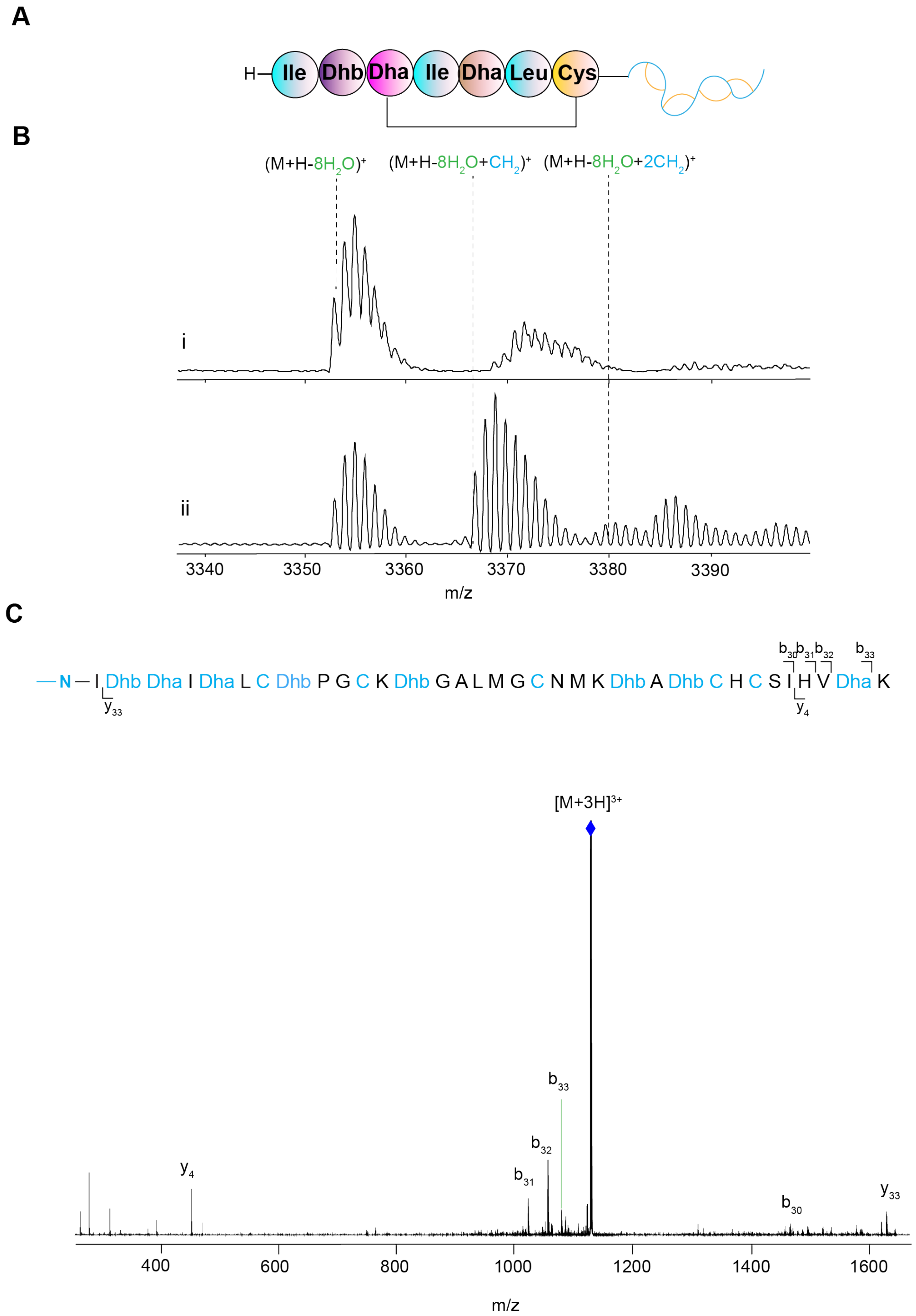

Figure S17. (A) Schematic representation of nisin. The N-terminal sequence of the peptide is described in detail, with a short-hand depiction of the C-terminal structure. **(B)** MALDI-TOF MS of nisin before CaoS$_C$ treatment (i) and after CaoS$_C$ treatment (ii). In (i), $(M + H – 8\ H_2O)^+$ calcd. *m/z* = 3352.5, obsd. *m/z*= 3352.6. In (ii), $(M + H – 8\ H_2O)^+$ calcd. *m/z* = 3352.5, obsd. *m/z*= 3352.8; $(M + H – 8\ H_2O + CH_2)^+$ calcd. *m/z* = 3366.5, obsd. *m/z*= 3367.8; $(M + H – 8\ H_2O + 2\ CH_2)^+$ calcd. *m/z* = 3380.5. **(C)** LC-ESI-QTOF MS−MS fragmentation pattern of the mono methylated nisin catalyzed by CaoS$_C$. For fragment masses, see Table S7.

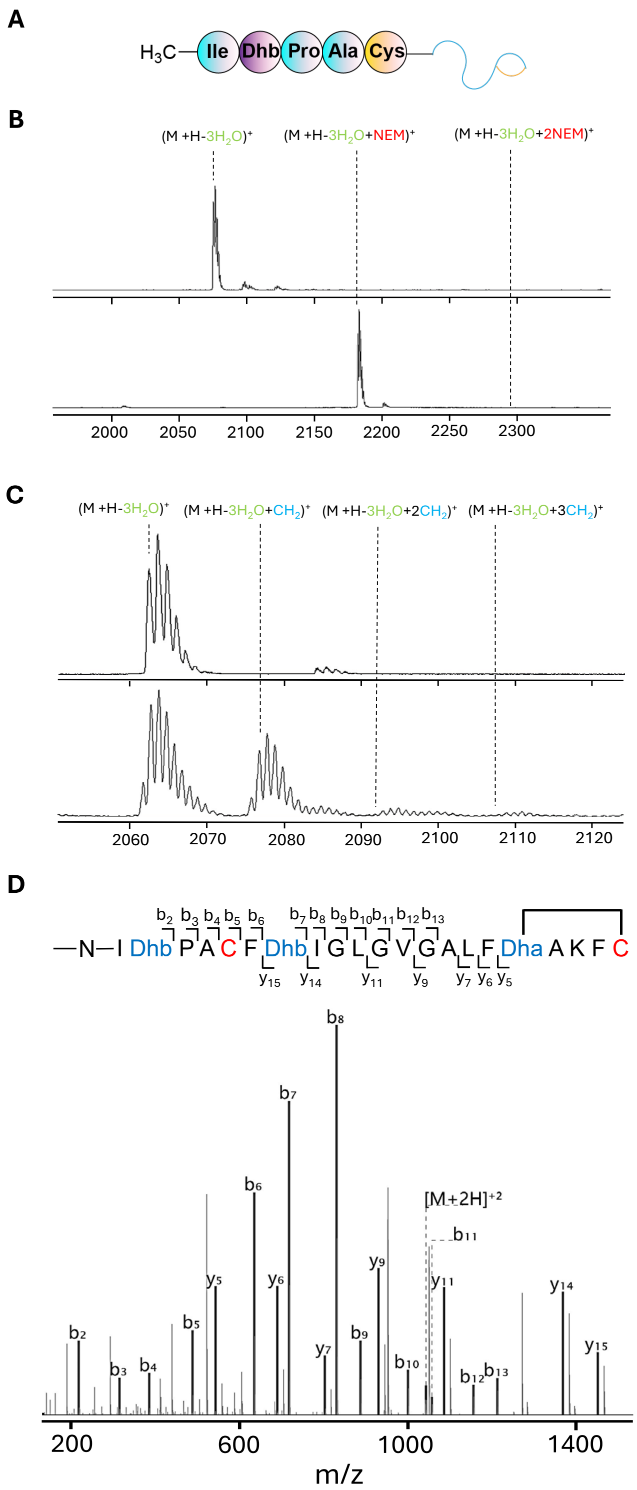

Figure S18. (A) Schematic representation of CylM-modified, CylA-digested His$_6$–CylL$_S$-(T1I). The N-terminal sequence of the peptide is described in detail, with a short-hand depiction of the C-terminal structure. **(B)** MALDI-TOF MS of the peptide described in (A) before NEM treatment (i) and after NEM treatment (ii). In (i), $(M + H - 3\ H_2O)^+$ calcd. *m/z* = 2062.1, obsd. *m/z*=2062.1. In (ii) $(M + H - 3\ H_2O + NEM)^+$ calcd. *m/z* = 2187.2, obsd. *m/z*= 2187.3; (M + H –

3 H_2O+ 2 NEM)$^+$ calcd. *m/z* = 2312.4. **(C)** MALDI-TOF MS of CylL$_S$-(T1I) before CaoS$_C$ treatment (i) and after CaoS$_C$ treatment (ii). In (i), (M + H - 3 H_2O)$^+$ calcd. *m/z* = 2062.1, obsd. *m/z*= 2062.1; In (ii), (M + H - 3 H_2O)$^+$ calcd. *m/z* = 2062.1; (M + H – 3 H_2O + CH_2)$^+$ calcd. *m/z* = 2076.1, obsd. *m/z*= 2076.2; (M + H – 3 H_2O+ 2 CH_2)$^+$ calcd. *m/z* = 2090.1; (M + H – 3 H_2O+ 3 CH_2)$^+$ calcd. *m/z* = 2104.1. **(D)** LC-ESI-QTOF MS−MS fragmentation pattern of the monomethylated CylL$_S$-(T1I) catalyzed by CaoS$_C$. For fragment masses, see Table S8.

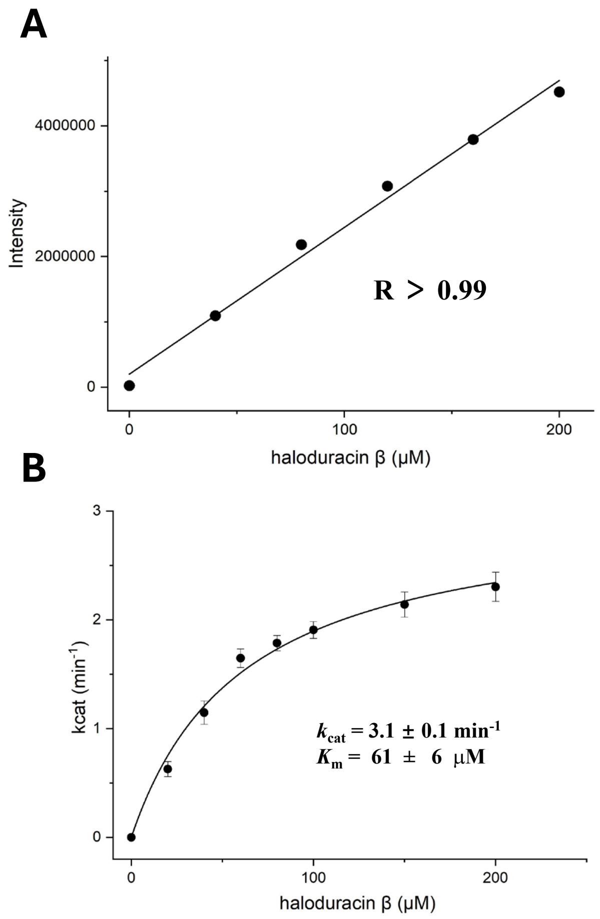

Figure S19. Kinetic analysis of methylation of haloduracin β by CaoS$_C$. (A) Intensity of the observed ion of haloduracin β as a function of peptide concentration showing linearity in the concentration range used. **(B)** Dependence of initial rates on substrate concentration as measured by substrate consumption using LC-MS from three independent experiments. A fit of the data to the Michaelis-Menten equation provided the kinetic parameters shown.

References

(1) Knerr, P. J., and van der Donk, W. A. Chemical synthesis and biological activity of analogues of the lantibiotic epilancin 15X, *J. Am. Chem. Soc.* **2012**, *134*, 7648-7651.
(2) Chalker, J. M., Gunnoo, S. B., Boutureira, O., Gerstberger, S. C., Fernández-González, M., Bernardes, G. J. L., Griffin, L., Hailu, H., Schofield, C. J., and Davis, B. G. Methods for converting cysteine to dehydroalanine on peptides and proteins, *Chem. Sci.* **2011**, *2*, 1666-1676.
(3) Aisporna, A., Benton, H. P., Chen, A., Derks, R. J. E., Galano, J. M., Giera, M., and Siuzdak, G. Neutral loss mass spectral data enhances molecular similarity analysis in METLIN, *J. Am. Soc. Mass Spectrom.* **2022**, *33*, 530-534.

(4) Montalbán-López, M., Scott, T. A., Ramesh, S., Rahman, I. R., van Heel, A. J., Viel, J. H., Bandarian, V., Dittmann, E., Genilloud, O., Goto, Y., Grande Burgos, M. J., Hill, C., Kim, S., Koehnke, J., Latham, J. A., Link, A. J., Martínez, B., Nair, S. K., Nicolet, Y., Rebuffat, S., Sahl, H.-G., Sareen, D., Schmidt, E. W., Schmitt, L., Severinov, K., Süssmuth, R. D., Truman, A. W., Wang, H., Weng, J.-K., van Wezel, G. P., Zhang, Q., Zhong, J., Piel, J., Mitchell, D. A., Kuipers, O. P., and van der Donk, W. A. New developments in RiPP discovery, enzymology and engineering, *Nat. Prod. Rep.* **2021**, *138*, 130 - 239.
(5) Letunic, I., and Bork, P. Interactive Tree Of Life (iTOL) v5: an online tool for phylogenetic tree display and annotation, *Nucleic Acids Res.* **2021**, W293-W296.
(6) Su, Y., Han, M., Meng, X., Feng, Y., Luo, S., Yu, C., Zheng, G., and Zhu, S. Discovery and characterization of a novel C-terminal peptide carboxyl methyltransferase in a lassomycin-like lasso peptide biosynthetic pathway, *Appl. Microbiol. Biotechnol.* **2019**, *103*, 2649-2664.
(7) Cooke, H. A., Guenther, E. L., Luo, Y. G., Shen, B., and Bruner, S. D. Molecular basis of substrate promiscuity for the SAM-dependent O-mNcsB1, involved in the biosynthesis of the enediyne antitumor antibiotic neocarzinostatin, *Biochemistry* **2009**, *48*, 9590-9598.
(8) Zallot, R., Oberg, N., and Gerlt, J. A. The EFI web resource for genomic enzymology tools: Leveraging protein, genome, and metagenome databases to discover novel enzymes and metabolic pathways, *Biochemistry* **2019**, *58*, 4169-4182.
(9) Gerlt, J. A., Bouvier, J. T., Davidson, D. B., Imker, H. J., Sadkhin, B., Slater, D. R., and Whalen, K. L. Enzyme Function Initiative-Enzyme Similarity Tool (EFI-EST): A web tool for generating protein sequence similarity networks, *Biochim. Biophys. Acta* **2015**, *1854*, 1019-1037.
(10) Liang, H., Lopez, I. J., Sánchez-Hidalgo, M., Genilloud, O., and van der Donk, W. A. Mechanistic studies on dehydration in Class V lanthipeptides, *ACS Chem. Biol.* **2022**, *17*, 2519-2527.
(11) McClerren, A. L., Cooper, L. E., Quan, C., Thomas, P. M., Kelleher, N. L., and van der Donk, W. A. Discovery and in vitro biosynthesis of haloduracin, a two-component lantibiotic, *Proc. Natl. Acad. Sci. U. S. A.* **2006**, *103*, 17243-17248.
(12) Bernardes, G. J., Chalker, J. M., Errey, J. C., and Davis, B. G. Facile conversion of cysteine and alkyl cysteines to dehydroalanine on protein surfaces: versatile and switchable access to functionalized proteins, *J. Am. Chem. Soc.* **2008**, *130*, 5052-5053.
